# Rapid Review and Meta-Meta-Analysis of Self-Guided Interventions to Address Anxiety, Depression, and Stress During COVID-19 Social Distancing

**DOI:** 10.3389/fpsyg.2020.563876

**Published:** 2020-10-28

**Authors:** Ronald Fischer, Tiago Bortolini, Johannes Alfons Karl, Marcelo Zilberberg, Kealagh Robinson, André Rabelo, Lucas Gemal, Daniel Wegerhoff, Thị Bảo Trâm Nguyễn, Briar Irving, Megan Chrystal, Paulo Mattos

**Affiliations:** ^1^School of Psychology, Victoria University of Wellington, Wellington, New Zealand; ^2^Instituto D'Or de Pesquisa & Ensino, Rio de Janeiro, Brazil; ^3^TAL Education Group, Beijing, China; ^4^Universidade de Brasília, Brasília, Brazil; ^5^Medical School, Institute of Psychiatry, Federal University of Rio de Janeiro, Rio de Janeiro, Brazil

**Keywords:** COVID-19, meta-analysis, self-guided interventions, depression, anxiety, culture, stress, subjective well-being

## Abstract

We conducted a rapid review and quantitative summary of meta-analyses that have examined interventions which can be used by individuals during quarantine and social distancing to manage anxiety, depression, stress, and subjective well-being. A literature search yielded 34 meta-analyses (total number of studies *k* = 1,390, *n* = 145,744) that were summarized. Overall, self-guided interventions showed small to medium effects in comparison to control groups. In particular, self-guided therapeutic approaches (including cognitive-behavioral, mindfulness, and acceptance-based interventions), selected positive psychology interventions, and multi-component and activity-based interventions (music, physical exercise) showed promising evidence for effectiveness. Overall, self-guided interventions on average did not show the same degree of effectiveness as traditional guided individual or group therapies. There was no consistent evidence of dose effects, baseline differences, and differential effectiveness of eHealth interventions. More research on the effectiveness of interventions in diverse cultural settings is needed.

## Impact Statement

Social distancing measures are effective in reducing viral spread in the current COVID-19 pandemic but have been shown to increase mental health burdens. These collateral effects are affecting large numbers of individuals globally, requiring urgent attention because of the strains on mental health providers struggling to provide adequate support for people in need. Although there are many self-help guidelines available online and via social media, it is unclear how effective these are. We provide a quantitative review of evidence-based practices that can be used by individuals at home or in confined physical environments during social distancing and quarantine to manage anxiety, depression, and stress. Given the likely continuation of social distancing measures in various parts of the world and shortages in mental health systems globally, our systematic review provides evidence on effective self-guided interventions, either as an initial stand-alone self-help intervention or while waiting for treatment.

## Introduction

What strategies can an individual adopt to maintain good mental health and reduce anxiety and stress during quarantines and physical distancing? Quarantines are psychologically taxing (Brooks et al., 2020), yet quarantines and physical distancing are core behavioral strategies for containing the spread of communicable diseases such as COVID-19. Levels of depression, anxiety, and psychological stress tend to be significantly elevated and can reach clinical levels in both disease survivors as well as the general population during pandemics. For example, Reynolds et al. (2008) reported that over 40% of quarantined Canadians in their sample reported high levels of worry. In the context of the COVID-19 pandemic, Wang et al. (2020) found that 28% of Chinese respondents reported clinically relevant levels of anxiety. Across both studies, over 50% of the sample indicated moderate to high levels of stress. The long-term psychological consequences of quarantine can last for months or possibly years (Brooks et al., 2020; Ho et al., 2020). The current COVID-19 (SARS-CoV-2) pandemic is affecting individuals globally at an unprecedented scale. Although widespread physical distancing measures appears effective in mitigating the spread of COVID-19 (Milne and Xie, 2020), the psychological ramifications of social distancing may result in increased levels of mental health problems in the near-term future.

Public mental health resources are finite, and the mental health services currently available are unlikely to cope with the emerging demands (Dong and Bouey, 2020; Duan and Zhu, 2020; Xiang et al., 2020). Addressing mental health needs within a physical distancing context is critical, given the possibility of continued movement restrictions in the near future to combat repeated outbreaks of SARS-CoV-2 (Kissler et al., 2020). To bolster available mental health services, while also reducing the likelihood of virus transmission, there has been a recent drive to convert usual face-to-face mental health treatments into an online or tele-health format.

However, while such modifications are no doubt vital, they represent only one building block of an organized mental health response, particularly when dealing with a pandemic such as COVID-19. Furthermore, even with increased use of tele-health measures by mental health providers, the shortage of trained professionals coupled with the increased demand on public health services highlights the need for effective and evidence-based self-guided therapeutic interventions (Duan and Zhu, 2020). The “*World Health Organization Service Organization Pyramid for an Optimal Mix of Services for Mental Health*” highlights self-care approaches (actions taken by individuals to improve their well-being) as an essential component of optimal mental health care (World Health Organization, 2003). In order to promote resilience and to appropriately manage the emerging mental health impacts of the ongoing COVID-19 pandemic, it is necessary to identify effective self-guided approaches to manage the psychological demands experienced during such outbreaks. Self-guided interventions can provide a first point of intervention for concerned individuals to alleviate anxieties, stress and worries, decrease negative mood and depressive symptoms, and increase positive psychological functioning and subjective well-being, either as a stand-alone intervention or while waiting for treatment. Although there are many self-help guidelines available online and via social media, it is unclear how effective these are and how well they are grounded in scientific evidence.

The goal of our rapid review is to provide a broad summary of the current evidence drawn from published meta-analyses in order to evaluate the effectiveness of self-guided therapeutic practices which can be implemented by individuals on their own, including during physical distancing and quarantine measures. We focused on published meta-analyses of randomized controlled trials (RCT) or experimental studies that evaluate the effectiveness of psychological interventions and strategies for a range of psychological outcomes. In particular, we focus on increasing subjective well-being (including life satisfaction, quality of life, happiness), and decreasing anxiety, depression, or stress as key outcomes. Critically, we screened all meta-analyses identified by our search parameters, but only summarize evidence from those meta-analyses which included self-guided conditions which can be performed by individuals alone without the guidance of trained health professionals. Our meta-analysis is more inclusive in scope because previous meta-analyses have: (a) typically focused on either a specific type of intervention or compared a small number of interventions without considering the wider range of possible interventions that might be beneficial; or (b) did not specifically consider the relevance and evidence of self-guided practices that could be performed by individuals alone. Thus, our primary aim is to provide a comparative summary of the available evidence of diverse psychological strategies that can inform recommendations by public health workers and psychologists, as well as be made available to the larger public. The COVID-19 pandemic affects populations of all nations, but interventions are often conducted with Western, industrialized and individualistic samples (Henrich et al., 2010), requiring more attention to cultural differences in effectiveness. We therefore evaluated whether the meta-analyses included in this review reported differences in treatment effectiveness for individuals from different cultural backgrounds. We focus on interventions that might be applicable in the current pandemic (and beyond), but explicitly stress that our data is not based on interventions conducted during the current COVID-19 pandemic. In order to provide actionable advice, we provide an electronic supplement containing selected self-guided exercises based on evidence gathered in this review. These exercises and tasks were selected with attention to possible applications across different cultural and economic contexts.

## Method

We performed a PsycInfo and MedLine search on March 22, 2020 to identify meta-analyses that have summarized RCTs or experimental studies that report the effectiveness of interventions on anxiety, depression, stress, or subjective well-being in human populations. The exact search terms and their combination are listed in Appendix A. The inclusion criteria for our review were: (a) quantitative meta-analysis of RCTs or experimental studies; (b) conducted with general populations, clinical or non-clinical samples, or samples selected for anxiety or depression symptoms; (c) the sample was on average 18 years or older; (d) measures of anxiety, depression, stress, or subjective well-being were included; (e) the meta-analysis included interventions that are self-guided or could be used by individuals without supervision or guidance by a trainer, therapist, or mental health professional; and (f) reported sufficient information on effect sizes. Where available, relevant moderator conditions were also extracted for further analysis. We decided to include anxiety and depression clinical samples due to the reported increase of anxiety and depression during quarantines (Brooks et al., 2020). The exclusion criteria for our review were: (a) clinical or patient populations other than individuals or groups with clinical anxiety and depression symptoms; (b) meta-analyses of group-based interventions; (c) meta-analyses of individual therapies or interventions led by or supervised/assisted by another person; (d) meta-analyses that did not clearly report on conditions in criteria a-c; (e) meta-analyses focusing exclusively on children or adolescents; (f) systematic reviews; (g) meta-analyses of cross-sectional or correlational studies; and (h) not published in a peer-reviewed English language journal. Regarding group and clinician-led interventions, we included meta-analyses if the authors tested delivery and application effects and found no significant differences between self-guided and other applications. If a meta-analysis examined those differences and reported differential effects for self-guided interventions, we only included those effect sizes relevant for self-guided interventions.

**Figure 1:**
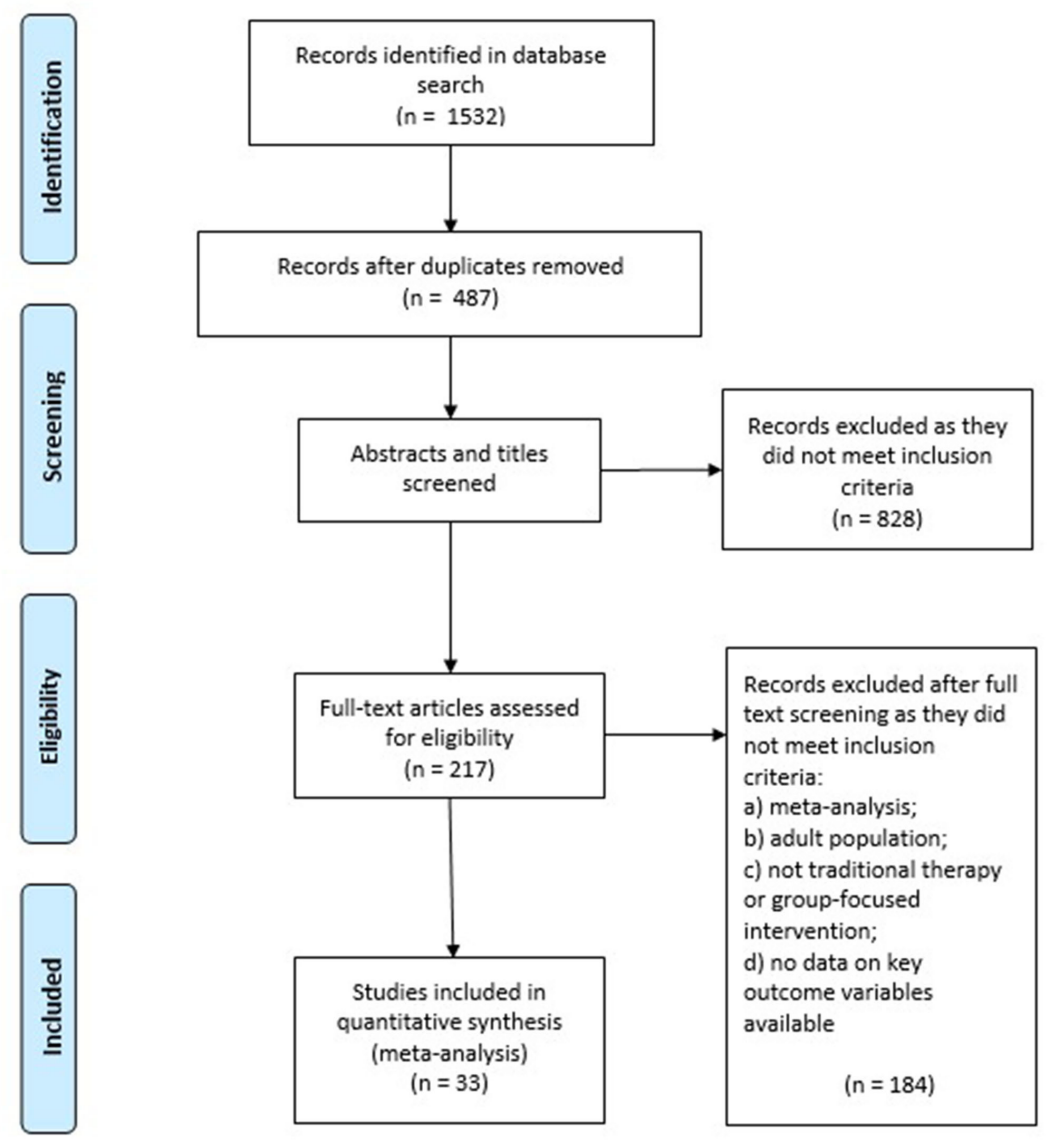
Prisma diagram.

We identified a substantial number of meta-analyses which examined the effectiveness of specific interventions, particularly for contemporary therapeutic approaches such as mindfulness and acceptance and commitment therapy (e.g., Hayes et al., 2012). Using all eligible meta-analyses may mean that identical primary studies might be included in a series of meta-analyses, leading to potential double-counting and duplication of effect sizes that would bias the overall patterns. To overcome this problem, we adopted the following strategies. First, we screened meta-analyses in a reverse temporal order, starting with the most recent meta-analyses per category. We then identified overlap in included primary studies between subsequent meta-analyses per outcome variable. If there was a 50% overlap or larger between two meta-analyses for a specific outcome variable, we included only the meta-analysis with the larger sample size. We still examined smaller meta-analyses to check if they reported moderator analyses of interest for our purposes, particularly the effectiveness of self-guided vs. other-guided or group interventions and differences in effectiveness between different cultural samples. As not all meta-analyses provided estimates for each of the outcomes of interest in this review, we repeated this process for each outcome variable. See Figure 1 for a PRISMA diagram of the selection process.

## Meta-Meta-Analysis Approach

We present the average effect size and 95% confidence interval as reported in the original meta-analyses in the form of a forest plot. The most common effect sizes are variations of the standardized mean difference (typically *d* or *g*), therefore, we use these metrics for plotting the effects. If no confidence interval was reported, only the mean effect size is displayed.

However, this visual display does not easily allow a statistical summary of the overall effect sizes. Therefore, we converted standardized mean differences into *r* and then z-transformed *r* (Rosenthal, 1991). As expected, the transformed effect size and the original effect size correlated *r* = 1.0. Inverse variance weights were calculated from sample sizes. If only the overall sample size was available, we used the average sample size per study to estimate sample sizes for subgroups. The average effect sizes per intervention category were computed using rma with REML estimation in the metafor package in R (Viechtbauer, 2010). We report the unstandardized regression weights.

## Results

### Participant Characteristics

Our sample of meta-analyses included 34 meta-analyses (total number of studies *k* = 1,390, *n* = 145,744). The majority of meta-analyses included general population samples, including working adults (meta-analysis *k* = 16). The second largest sample group were mixed general population and clinical samples (meta-analysis *k* = 11). Purely clinical samples were included in 4 meta-analyses and students were the primary population in 3 meta-analyses. All but one meta-analysis (Dickens, 2017) exclusively focused on adult populations. Only 5 meta-analyses explicitly reported and tested cross-cultural differences.

### Qualitative Review of Published Meta-Analyses

We used two approaches to evaluate the relative effectiveness of self-guided interventions. First, we used Cohen (1988) effect size benchmarks to specify small (*d* = 0.2), medium (*d* = 0.5), and large (*d* = 0.8) effect sizes of standardized mean differences. This first allows us to assess the overall effectiveness of self-guided interventions compared to control interventions (typically, waitlist, or active control groups). Second, we compared the overall effect sizes of self-guided interventions against effectiveness benchmarks of traditional clinician-guided psychotherapy for reducing depression symptoms (Cuijpers et al., 2020). The overall effect size of traditional clinician-guided psychotherapy on depression was *g* = 0.72 (*k* = 385), with Cognitive-Behavioral Therapy (CBT) showing an effectiveness of *g* = 0.73 (*k* = 205); Behavioral Activation Theory *g* = 1.05 (*k* = 21), and third-wave therapies (including Acceptance and Commitment Therapy [ACT] and Mindfulness-Based Interventions [MBI]) an effectiveness of *g* = 0.85 (*k* = 19).

#### Effectiveness Across Types of Self-Guided Interventions

Table 1 shows an overview of the meta-analyses included in this review. The large majority of psychological intervention meta-analyses that were eligible to be included in our review consisted of meta-analyses of clinical psychology therapy-derived interventions (*k* = 17), which encompass self-guided CBT, ACT, and MBI, as well as diverse Positive Psychology-based interventions (*k* = 8).

**Table 1:** Overview of meta-analytical findings.

**Article**	**Population**	**Type of interventions**	**Control groups**	**Data quality**	**Data base *n* (*k*)**	**Evidence of effectiveness of self-guided interventions**	**Effect sizes**	**Dose effects**	**Base line effects**	**Cultural differences**
Blanck et al. (2018)	Primarily student	Mindfulness (guided; audio-tapes)	CT and RCT with active and inactive controls	Tulder Quality Assessment scale mean = 5.59 (SD = 1.56); 10 studies had high quality, 5 studies judged low quality (out of 18); Evidence of publication bias (funnel plot, Egger regression)	*n* = 1,150 (*k* = 18)	No significant difference between guided and self-administered interventions	Anxiety: *g* = 0.39 [0.22, 0.56] (Overall compared to inactive), *g* = 0.27 [0.03, 0.50] (Overall compared to active); Depression: *g* = 0.41[0.19, 0.64] (Overall compared to inactive), *g* = 0.27[−0.04, 0.58] (Overall compared to active)	Practice time and duration of individual sessions did not show significant effects	NA	NA
Cavanagh et al. (2014)	General population	Self-help mindfulness and acceptance-based interventions	RCT with active and inactive control	Jadad score: on average medium quality; publication bias for anxiety, but not depression (funnel plot)	*n* = 2,286, (*k* = 15)	Guided interventions show larger effect (*post-hoc* analysis)	Anxiety: *g* = 0.34 [0.10, 0.57], Depression: *g* = 0.37 [0.19, 0.56]	Self-help interventions: The mindfulness and/or acceptance components resulted in a significantly higher level of mindfulness/acceptance skills and significantly lower levels of anxiety and depressive symptoms than control conditions, with small to medium effect sizes	NA	NA
Chu and Mak (2020)	Clinical and general population	Mindfulness (including meditation, Loving-kindness)	RCT with active and inactive control	RCTs showed medium quality on average. Higher-quality studies showed significantly smaller ES's. Evidence of publication bias (Egger regression)	*n* = 912 (*k* = 11, RCT's only)	Online studies showed higher ES than group (but small number of valid comparisons)	SWB (Satisfaction with life): *g* = 0.53 [0.26, 0.80] (RTCs)	NA	No difference between clinical and general populations	No significant difference between regions (North America; Europe/Australia; Asia; other)
Conn (2010)	General population	Physical activity	Experimental studies (including quasi-experiments and pre-post comparisons)	Random assignment shows significantly smaller effects	Control group designs: *n* = 1,081 (*k* = 22); Pre-post design: *n* = 3,420 (*k* = 45)	Individual vs. group training not significantly different	Depression: *d =* 0.52 [0.28; 0.77] for control group designs; *d =* 0.47 [0.38; 0.56] for pre-post designs	home exercise less effective than fitness center; more training per week less effective; shorter trainings more effective	NA	NA
Cregg and Cheavens (2020)	Clinical and general population	Gratitude	RCT with active and inactive control	Cochrane: majority of studies was classified as medium to high risk of bias; bias rating did not significant moderate ES overall (studies where participants were aware of condition had larger pooled ES compared to blinded/insufficient information studies); all outcomes are adjusted for unreliability. Possibility of publication bias (larger ES with smaller *n*)	*n* = 3,675 (*k* = 27)	No difference between online vs. offline activities	Anxiety: *g* = −0.16 [−0.38, 0.05]; Depression: *g* = −0.17 [−0.24, −0.10] (outliers excluded)	Duration (days, weeks) and compliance do no moderate ES	Level of depression does not moderate ES	NA
Cuijpers et al. (2011)	Clinical samples	Self-guided interventions (mainly CBT)	RCT with active and inactive control	Cochrane: acceptable level of bias (but no blinding); no evidence of publication bias (Egger regression)	*n* =1,362 (*k* = 7)	Self-guided interventions are effective compared to control; personal contact vs. complete self-help did not moderate effect size	Depression: *d* = 0.28 [0.14; 0.42]	NA	NA	
Curry et al. (2018)	General population	Kindness (other focused; excluding loving-kindness)	Experimental studies	No quality rating; no evidence of publication bias (funnel plot)	*n* = 4,045 (*k* = 27)	NA	SWB: *d* = 0.28 [0.16, 0.41]	NA	No differences between socially anxious and other populations	NA
Davies et al. (2014)	Clinical and non-clinical samples	Multicomponent online interventions	RCT with active and inactive control	Cochrane: Moderate quality on average	*n* = 1,480 (*k* = 17)	NA	Inactive control: Anxiety: *g* = −0.56 [−0.77; −0.35]; Depression: *g* = −0.43 [−0.63; −0.22]; Stress: *g* = −0.73 [−1.27; −0.19] Active control: Anxiety: *g* = −0.18 [−0.98; 0.62]; Depression: *g* = −0.28 [−0.75; −0.20]; Comparison intervention: Anxiety: *g* = −0.10 [−0.39; −0.18]; Depression: *g* = 0.33 [−0.43; 1.09]	NA	NA	NA
de Witte et al. (2019)	General population	Music activities and music therapy	RCT with active and inactive control	Quality rated and no evidence of publication bias (funnel plot)	*n* = 6,800 (*k* = 79)	No significant difference between music therapy and self-guided music activities	Anxiety: *g* = 0.55; Stress: *g =* 0.51	No effect of frequency or duration	No differences between surgery, non-medical, or polyclinical procedures	No difference between Western or Non-Western samples
Deady et al. (2017)	General population	eHealth (8 CBT; 1 ACT, 1 self-help emails)	RCT with active and inactive control	Downs and Black checklist: Fair to good quality; no evidence of publication bias (Egger regression)	*n* = 4,522 (*k* = 10)	NA	Anxiety: *d* = 0.31 [0.10; 0.52]; Depression: *d* = 0.25 [0.09; 0.41]	NA	No difference between general and indicated/selected populations	NA
Dickens (2017)	General population (including children)	Gratitude	Experimental (including quasi-experimental), comparing gratitude to neutral, negative, and positive intervention	NA. Evidence that negative interventions (focusing on hassles etc.) produce significantly larger ES	*n* = 3,351 (*k* = 38)	NA	Gratitude vs. Neutral: Depression: *d* = 0.13; SWB (life satisfaction): *d* = 0.17; Stress: *d* = 0.04; Gratitude vs. Positive: Depression: *d* = 0.02; SWB (life satisfaction): *d* = 0.03; Stress: *d* = −0.03	NA	NA	NA
Firth et al. (2017)	Clinical and non-clinical samples	eHealth	RCT with active and inactive control	Cochrane: most show lack of blinding; No evidence of publication bias (funnel plot)	*n* = 3,414 (*k* = 18)	eHealth interventions with “in-person” (i.e., human) compared to without feedback had small, non-significant effects on depressive symptoms; in-app feedback applications showed slightly greater ES compared to no in-app feedback; self-contained smartphone apps showed slightly larger ES compared to non-self-contained interventions (*p* = 0.07)	Inactive control: Depression: *g* = 0.56 [0.38; 0.74]; Active control: *g* = 0.22 [0.10; 0.33]	Length (in weeks) showed a trend to reduce effectiveness	Mild-to-moderate depressive groups showed larger improvement; no significant ES for samples with major depressive disorder, bipolar disorder, and anxiety disorders (but possible lack of power)	NA
Frattaroli (2006)	Clinical and general population	Expressive writing	RCT with neutral or waitlist control	Mean quality rating = 2.94 (scale 0–4); higher quality studies show smaller psychological health effect (strongest impact for participant expectation of study benefit); larger *n* was associated with weaker effects (possible publication bias)	*n* = 8,533 (*k* = 112)	Larger ES when expressive writing was conducted at home and in private settings	Anxiety: *r* = 0.03 [−0.09; 0.19]; Depression: *r* = 0.04 [−0.11; 0.16]; SWB: (Satisfaction with life) *r* = 0.03 [0.01; 0.08]; Stress *r* = 0.02 [−0.02; 0.08]	Trend for larger ES with more than 3 sessions; no effect of length of disclosure or spacing of sessions	Studies with participants with a history of trauma or stressors did not moderate ES; writing about more recent trauma showed stronger effect	No effects for proportion of ethnic minorities
Heekerens and Eid (2020)	General population	Positive psychology intervention (best-possible-self intervention)	RCT with active control group	Cochrane:	*n* = 4,462 (*k* = 34)	NA	Depression: *g* = −0.09 [−0.23; 0.06]; SWB (Life satisfaction): *g* < 0.01 [−0.09; 0.09]	NA	NA	NA
Hendriks et al. (2018)	Clinical and non-clinical samples	Positive psychology interventions	RCT with active and inactive control	Cochrane: mean quality score 1.79 on 0–6 scale	*n*= 3,009 (*k* = 28)	Self-guided interventions showed no effect (compared with group studies, but difference not significant)	Anxiety: *g* = 0.95 [0.28; 1.61]; Depression: *g* = 0.62 [0.19; 1.05]; SWB: *g* = 0.48 [0.24; 0.72]	Longer interventions showed larger ES	No significant difference	Non-western samples only
Hendriks et al. (2020)	Clinical and non-clinical samples	Positive psychology interventions	RCT with active and inactive control	Cochrane: 26% (13 studies) had high quality, average study *M* = 3.2 on 0–6 scale; Low quality studies show higher effect than moderate quality studies; funnel plot and Egger regression show some inconsistent evidence of publication bias	*n* = 6,141 (*k* = 50)	No statistically significant difference between individual, self-help, and group studies	Anxiety: *g* = 0.35 [0.23; 0.48]; Depression *g* = 0.32 [0.13; 0.51]; SWB: *g* = 0.34 [0.18; 0.50]; Stress: *g* = 0.35 [0.03; 0.66]	Inconsistent duration and session effects	No difference between clinical and general populations	Non-Western samples show significantly larger ES compared to Western samples
Huang et al. (2018)	Students	Diverse interventions	RCT with active and inactive control	CONSORT rating: moderate compliance	*n* = 3,602 (*k* = 51)	Easy to disseminate interventions (less guidance, etc.) showed smaller effects	Anxiety overall: *g* = 0.48 [−0.62; −0.34]; For mixed/other interventions: *g* = −0.84 [−1.19, −0.49]; CBT *g* = −0.39 [−0.55; −0.22]; Mindfulness–based: *g* = −0.49 [−0.84, −0.15]; Depression overall effects: *g* = −0.60 [−0.74, −0.46]; For mixed/other interventions: *g* = −0.76 [−1.19, −0.32], CBT: *g* = −0.59 [−0.72, −0.45]; Mindfulness-based: *g* = −0.52 [−0.88, −0.16]; Attention/perception modification: *g* = −0.46 [−1.06, 0.13]	Longer interventions showed larger ES	NA	Effects for depression vary by region (in order of effectiveness): Asia > Australia > North America > Europe; no effects for anxiety
Karyotaki et al. (2017)	Clinical samples	self-guided internet-based CBT	RCT with active and inactive control	Cochrane: overall low risk of bias (but no blinding); evidence of publication bias (Egger regression)	*n* = 3,876 (*k* = 16)	Self-guided interventions are effective compared to control; adherence increases effectiveness	Depression: *g* = 0.27 [0.17, 0.37]	No significant effect for treatment duration	No baseline effects	NA
Kirby et al. (2017)	General adult population	Compassion-based interventions (incl. loving kindness)	RCT with active and inactive control	Cochrane: most studies show low quality (blinding, reporting, attrition); funnel plot suggested weak evidence of publication bias	*n* = 1,285 (*k* = 20)	NA	Anxiety: *d* = 0.49 [0.30–0.68]; Depression: *d* = 0.64 [0.45–0.82]; SWB: *d* = 0.51 [0.30–0.63] (relative to waitlist); Anxiety: *d* = 0.42 [0.19; 0.64]; Depression: *d* = 0.62 [0.44-0.80]; SWB: *d* = 0.48 [0.28–0.67] (active control).	NA	NA	NA
Koydemir et al. (2020)	General population	Positive psychology interventions	RCT with active and inactive controls	No quality rating; Funnel plot suggests some publication bias	*n* = 16,085 (*k* = 68)	No statistically significant difference between self vs. trainer guided interventions; technologically assisted interventions significantly less effective than traditional interventions	SWB: *d* = 0.22	Duration effects significant (longer duration more effective)	NA	NA
Ma et al. (2019)	University Students	Mindfulness training and ACT	RCT with active and inactive control	Cochrane: 20% of studies showed high risk of bias (but study quality was not a significant moderator); evidence of publication bias (smaller n shows stronger effect)	*n* = 2,472 (*k* = 22)	Method of delivery had no significant effect	Depression: *g* = 0.52 [0.39, 0.65]	Weekly delivery more effective than more frequent training, inconsistent effects of duration (in weeks)	Indicated MBIs showed stronger effects than universal MBIs, but no difference with selective MBIs	NA
Malouff and Schutte (2017)	Clinical and general population	Optimism training (mostly best possible self and self-compassion)	RCT with active and inactive control	Funnel plot suggests some positive bias	*n* = 3,319 (*k* = 29)	Online studies showed weaker effect than in-person interventions	SWB (Optimism): *g* = 0.51 [0.36, 0.66] (waitlist control) SWB (Optimism): *g* = 0.23 [0.09, 0.37] (active control)	In-person intervention hours showed negative effect on ES (longer sessions less effective)	No difference for healthy vs. identified problem sample	NA
Massoudi et al. (2019)	Clinical population (anxiety, depression)	eHealth	RCT with active control group	Cochrane: Low risk of bias for 46.7% of trials, with high risk for 29.5%. No evidence of publication bias (symmetric funnel plot)	*n* = 4,183 (*k* = 14)	NA	Depression: *g* = −0.19 [−0.31, −0.06]	NA	NA	NA
O'Connor et al. (2018)	Clinical and non-clinical samples	eHealth third wave treatments (9 ACT, remainder mixture of CBT, mindfulness and others)	RCT with active and inactive control	Cochrane: moderate level of bias; bias is associated with larger ES; weak evidence of publication bias overall (funnel plot)	*n* = 3,176 (*k* = 21)	Therapist guidance did not significantly moderate ES	Inactive control: Anxiety: *g* = 0.32 [0.09, 0.56]; Depression: *g* = 0.52 [0.26, 0.77] Active control: Anxiety: *g* = 0.31 [0.07, 0.54]; Depression: *g* = 0.29 [0.14, 0.44] Comparison intervention: Anxiety: *g* < 0.01 [−0.16, 0.17]; Depression: *g* = −0.02 [−0.18, 0.15]	Number of intervention sessions did not moderate ES	No statistical difference between clinical vs. non-clinical populations	NA
Panteleeva et al. (2017)	General population	Music listening	RCT with active and inactive control	CONSORT rating: Low quality on average	*n* = 792 (*k* = 21)	NA	Anxiety: *d =* −0.30 [−0.55, −0.04]	NA	NA	NA
Pavlacic et al. (2019)	Clinical and general population	Expressive writing	Experimental (including pre–post studies)	No evidence of publication bias, but low power in *post-hoc* power analyses	*n* = 1,581 (*k* = 53)	NA	SWB (Quality of Life): *d = –* 0.01 [−0.16, 0.13]	*Post-hoc* analyses suggest that short term intervals show positive ES, longer time intervals show negative ES (but low power)	NA	NA
Reinhold et al. (2018)	General population (no PTSD diagnosis)	Expressive writing (emotional, personal topic)	RCT with active and inactive control	Cochrane analysis: quality not correlated with ES; removed one study with incorrect reporting	*n* = 4,009 (*k* = 39)	NA	Depression: *g* = −0.09 [−0.15, −0.02]	Higher number of writing sessions and specific writing topic (vs. general) showed higher ES	No effect of clinical vs. non-clinical samples, depression score at pre-test	NA
Slemp et al. (2019)	Working adults	Mindfulness-based work interventions (ACT included; yoga excluded)	Intervention based (including quasi-experimental)	Down and Black: overall poor quality. No effect of data quality on ES; evidence of publication bias (Egger regression)	*n* = 6,044 (*k* = 119)	Self-guided interventions where as effective as other guided interventions (*p* = 0.077)	Anxiety: *d* = 0.58 [0.37, 0.79]; Depression: *d* = 0.42 [0.24, 0.59]; Stress: *d* = 0.47 [0.35, 0.58]	No dose effects for duration (weeks) or number of sessions	NA	NA
Spijkerman et al. (2016)	General population	Online administered MBIs	RCT	Jadad scale and Cochrane: most studies (*k* =10) medium quality; Evidence of publication bias (funnel plot)	*n* = 2,360 (*k* = 15)	For stress: interventions supported by therapists produced larger effects than online only interventions; no differences found for anxiety, depression and well-being.	Anxiety: *g =* 0.19 [−0.06, 0.43] (Self–help only); *g =* 0.22 [0.05, 0.39] (Overall) Depression: *g =* 0.29 [0.03, 0.55] (Self-help only); *g =* 0.29 [0.13, 0.46] (Overall); Stress: *g =* 0.19 [−0.01, 0.38] (Only self-help); *g =* 0.51[0.26, 0.75] (Overall); Well-being: *g =* 0.31 [0.11, 0.52] (Self-help only); *g =* 0.23[0.09, 0.38] (Overall)	For stress: more sessions had stronger effect (when excluding outliers, this effect disappears)	No differences between general and groups with psychological problems	NA
Stratton et al. (2017)	Working adults	eHealth interventions (CBT, mindfulness, stress management)	RCT with waitlist control	Down and black ratings; evidence of publication bias (funnel plot, Egger regression)	*n* = 2,922 (*k* = 23)	Guided eHealth interventions show higher ES than unguided ones	Overall effects – Anxiety: *g* = 0.21; Depression: *g* = 0.25; Stress: *g* = 0.30	NA	Targeted populations (compared to untargeted) showed stronger ES overall (mainly driven by target effects for Stress Management on stress outcomes; no effect for CBT interventions)	NA
Strohmaier (2020)	Clinical and general population	MBCT/MBSR and other Mindfulness-based practices	RCT with active or inactive controls	Cochrane: Only five studies showed low risk of bias	*n* = 15,971 (*k* = 203)	No significant effects of the number of face-to-face sessions or contact hours	Compared to inactive controls: Anxiety: *d* = −0.49 [−0.59, −0.38]; Depression: *d* = −0.60 [−0.70, −0.50]; Stress: *d* = −0.73[−1.00, – 0.46] (Post–program). Compared to active controls: Anxiety: *d* = −0.16 [−0.26, −0.05]; Depression: *d* = −0.20 [−0.30, −0.11]; Stress: *d* = −0.32 [−0.61,−0.04]	Immediately post-program no dose response differences, but at 1–4 months follow-up shows inconsistent dose effects (e.g., home practice, intensity, facilitator contact)	No effect of baseline differences	NA
Vonderlin et al. (2020)	Working adults	Mindfulness work interventions (at least 2 h; at least 50% mindfulness practice; ACT and yoga included)	RCT with active and inactive control	Cochrane: low risk of bias; some evidence of publication bias for stress (funnel plot)	*n* = 5,161 (*k* = 56)	No effect of method of delivery (self-guided/online vs. in-person delivery)	SWB (Life satisfaction): *g* = 0.68 [0.24, 1.12]; Stress: *g* = −0.66 [−0.88, −0.44]	Program attendance hours increased SWB (but not duration in weeks)	NA	NA
Weisel et al. (2019)	General population	Multicomponent mobile health apps	RCT with active and inactive control	Cochrane: 53% (10/19) exhibit bias in at least three domains	*n* = 3,681 (*k* = 19)	Standalone smartphone apps	Anxiety: *g* = 0.43 [0.19; 0.66]; Depression: *g* = 0.34 [0.18, 0.49] (overall) Anxiety: *g* = 0.49 [0.27, 0.71]; Depression: *g* = 0.41 [0.24-0.59] (waitlist control)	Follow-up assessments were not examined	NA	NA
Yang (2018)	Clinical groups	Computer-Mediated Support Groups	RCTs and one-group pre-test post-test design	Possibility of publication bias (funnel plot, Egger regression)	*n* = 7,582 (*k* = 43)	Presence vs. absence of facilitator did not moderate ES	Depression: *d* = 0.32 [0.22; 0.43]	Group size was significant: larger online groups less effective	NA	NA

*if no 95% confidence intervals are included, they were not reported in the original meta-analysis*.

##### Self-guided therapy-derived interventions

A large number of studies used therapy-derived interventions including CBT, MBI, and ACT, and showed small to medium effect sizes for reducing anxiety, depression, and stress. Effects for subjective well-being in some analyses showed moderate to large effect sizes (Chu and Mak, 2020; Vonderlin et al., 2020). When compared to active control groups instead of non-active controls or waitlist groups, effect sizes typically diminished but remained statistically significant (e.g., Deady et al., 2017; O'Connor et al., 2018). Overall, the self-guided effect sizes tended to be lower than the effectiveness of traditional clinician-guided therapies, but clearly showed an effectiveness over and above active control groups (e.g., Spijkerman et al., 2016; Stratton et al., 2017). Other meta-analyses found no difference for self-guided compared to clinician-guided interventions (e.g., in general: Spijkerman et al., 2016; O'Connor et al., 2018 found no difference for anxiety and depression). Based on these meta-analyses, self-guided therapy-derived interventions are recommended to improve well-being during isolation.

##### Positive psychology-based interventions

Positive psychology-based interventions are typically focused on positive functioning, including interventions focusing on optimism, gratitude, or kindness. There is a somewhat older literature on expressive writing (Pennebaker, 1997) which we included here for convenience purposes. Overall, the effect sizes of positive psychology-based interventions were typically small and appear even more strongly affected by the type of control group than therapy-derived interventions (for a particularly striking example, see Dickens, 2017). Some of the positive psychology gratitude interventions differ by the focus of the intervention: either self- or interpersonally-oriented gratitude. These differences appear to be similarly effective (e.g., Cregg and Cheavens, 2020). Cregg and Cheavens (2020) found online compared to off-line applications equally effective, whereas Koydemir et al. (2020) reported greater effectiveness of non-technologically mediated interventions. Hendriks et al. (2020) reported that web-based interventions were as effective as online positive psychology apps. Expressive writing interventions showed the smallest effect sizes overall in this group, whereas compassion and kindness-based interventions showed moderate effect sizes in some meta-analyses (Kirby et al., 2017). Based on these meta-analyses, expressive writing interventions are the least effective, whereas gratitude, especially compassion-based interventions, could be recommended to improve well-being during quarantine and isolation.

##### Other activity-based interventions

Other activity-based interventions comprised a broad category including various physical exercise, arts, and music-based activities. Physical exercise showed weak effects overall in improving subjective well-being, with slightly larger effects for reducing depression (Conn, 2010). Music-based interventions also showed weak to moderate effects in reduced anxiety and stress levels (Panteleeva et al., 2017; de Witte et al., 2019). Therefore, activity-based interventions, including music and physical exercise, show small to moderate effects and could be recommended to improve mental health during isolation.

##### Multicomponent online and app-interventions

Multicomponent online and app-based interventions showed small to moderate effects, with diminished effects when compared to standard therapeutic interventions (see for example, O'Connor et al., 2018). Overall, their effectiveness was considerably smaller than similar non-online interventions (e.g., Malouff and Schutte, 2017) or standard in-person therapies. The relative effectiveness of online only compared to smartphone-based apps remains unclear (Firth et al., 2017; Weisel et al., 2019), and thus no clear guidance is available.

#### Dose Effects

Dose effects, or the extent to which people are practicing or using a specific activity, are important to consider when recommending self-guided practice. These might include total practice time, or duration of individual interventions. Several meta-analyses examined the effectiveness of interventions at different dose levels. For therapy- derived interventions, Blanck et al. (2018) and O'Connor et al. (2018) found no dose effects for self-guided CBT-based interventions. In contrast, Firth et al. (2017) reported a reduction (although not statistically significant) in the effectiveness of longer-term smartphone-based interventions, whereas Huang et al. (2018) reported greater effectiveness of longer ACT and CBT-based interventions on depression. Strohmaier (2020), Spijkerman et al. (2016, after removing outliers), and Slemp et al. (2019) found no difference in dose effects for MBI-based interventions. Focusing on life satisfaction, Vonderlin et al. (2020) reported greater life satisfaction was correlated with higher number of mindfulness-based practice hours, whereas Ma et al. (2019) reported inconsistent dose effects for mindfulness-based interventions in students, with a slight increase in effectiveness for weekly practices compared to more frequent sessions.

Positive psychology interventions also showed inconsistent dose effects. Hendriks et al. (2020) reported inconsistent patterns for interventions of more or less than 8 weeks for different outcome variables. Similarly, for expressive writing exercises, the dose effects are inconsistent across the studies that reported them (Frattaroli, 2006; Malouff and Schutte, 2017; Reinhold et al., 2018). For physical exercise, a meta-analysis by Conn (2010) found that home-based unsupervised exercise was less effective than unsupervised exercise in fitness centers. The same meta-analysis also suggested that shorter training overall might be more effective in improving depressive symptoms. de Witte et al. (2019) reported no significant dose effects for music interventions. Examining the overall pattern, dose effects appear inconsistent and no clear guidance is available about optimal levels of practice.

#### Baseline Effects and Applicability for Clinical Populations

One important concern in recommending self-guided interventions is whether these interventions are applicable for populations experiencing clinically relevant symptoms. In particular, a specific intervention may show no effect or an adverse effect in clinical populations, making the intervention unsafe for such populations. Hence, we investigated whether meta-analyses examined baseline effects of anxiety, depression, or stress on effectiveness, or directly compared the effectiveness between clinical and non-clinical populations.

Among therapy-derived interventions, several meta-analyses found no significant baseline effects or difference between clinical and non-clinical samples (Spijkerman et al., 2016; Deady et al., 2017; O'Connor et al., 2018; Strohmaier, 2020). For positive psychology interventions, several meta-analyses also found no difference between clinical and non-clinical samples (Frattaroli, 2006; Reinhold et al., 2018; Cregg and Cheavens, 2020; Hendriks et al., 2020). Only Pavlacic et al. (2019) reported larger effect sizes for groups with a Post-Traumatic Stress Disorder (PTSD) diagnosis compared to non-PTSD groups. No difference between clinical (labeled socially anxious) and non-clinical populations was found for kindness-based interventions (Curry et al., 2018). Taken together, this suggests that therapy- derived and positive psychology interventions in general could be recommended to populations irrespective of their depression or anxiety levels or clinical diagnosis status. For music interventions, de Witte et al. (2019) reported no differences between different populations in terms of effectiveness. For generic smart-phone applications (including a large number of clinical therapeutic approaches), Firth et al. (2017) reported that effectiveness of these apps was better for individuals diagnosed with mild-to-moderate levels of depression, but groups diagnosed with major depressive disorder, bipolar disorder, and anxiety disorder showed no significant improvement when using these applications. However, these sample sizes were typically small and might have been too small to show the effectiveness of these eHealth apps. Overall, clinical status or level of anxiety or depression do not exert a strong influence on the effectiveness of these self-guided interventions. This is encouraging news to support the wide-spread recommendation of these self-guided interventions in general, in the absence of immediate clinical guidance or supervision.

#### Cross-Cultural Applicability

The current COVID-19 pandemic is affecting all countries. For this reason, we also examined the extent to which the interventions might be applicable and effective in different cultural regions. Unfortunately, only a small number of meta-analyses (*k* = 5) attended to possible cultural differences in the effectiveness. Chu and Mak (2020) found no significant differences in mindfulness-based interventions between world regions and de Witte et al. (2019) comparing the effectiveness of music interventions reported no differences between Western and non-Western samples. In contrast, Hendriks et al. (2020) compared positive psychology interventions and reported larger effect sizes in non-Western compared to Western samples. However, it is unclear whether these comparisons might be confounded by other study characteristics. An earlier meta-analysis by Hendriks et al. (2018) only focused on non-Western interventions and reported low quality studies. Overall, it is noteworthy that there are relatively few high-quality studies available that have examined the effectiveness of self-guided psychological interventions in samples beyond Western Europe, North America, and Australia.

#### Contextualizing the Evidence-Base Against Excluded Intervention Types

We were unable to include any studies that exclusively looked at yoga or meditation because meta-analyses of these studies always included group settings or guidance by a trainer or clinician. Based on our inclusion criteria, we were unable to include them in our review. However, the evidence from recent meta-analyses suggests that these interventions are effective for anxiety, depression, and broader mental and physical health (see for example, Sedlmeier et al., 2018; Zoogman et al., 2019) and clinical network meta-analyses attest to their safety and effectiveness (Chen and Shan, 2019). A further advantage of these types of interventions is that they seem to show higher effectiveness in non-Western populations (e.g., Zoogman et al., 2019). Given the wide availability of online yoga and meditation sessions/apps and the overall effectiveness of guided yoga and meditation sessions *in situ*, we could cautiously recommend the practice of yoga and meditation for improving mental health during quarantine and social distancing conditions.

### Meta-Meta-Analysis of the Effectiveness of Self-Guided Interventions

**Figure 2:**
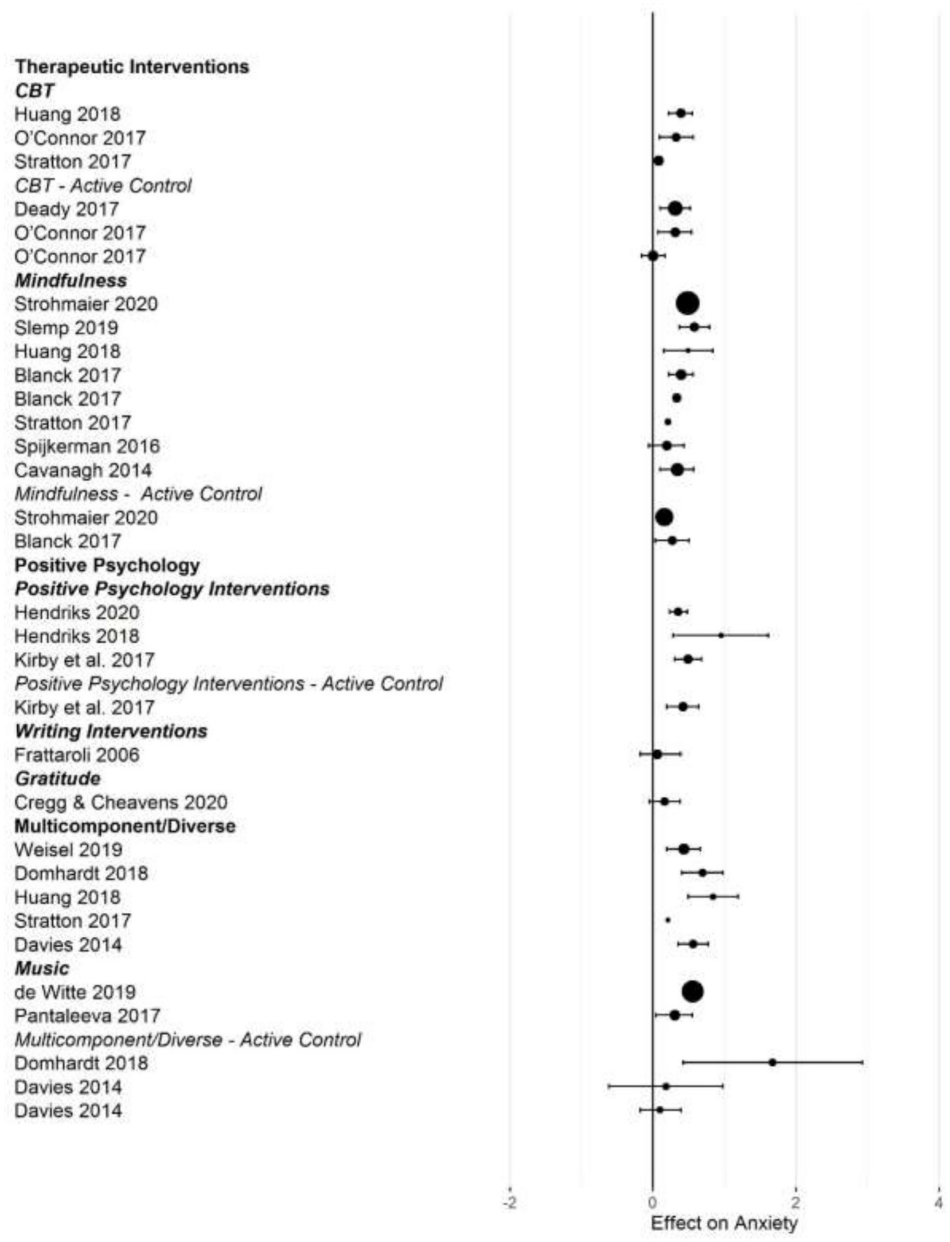
Forest plot of intervention effects on anxiety.

**Figure 3:**
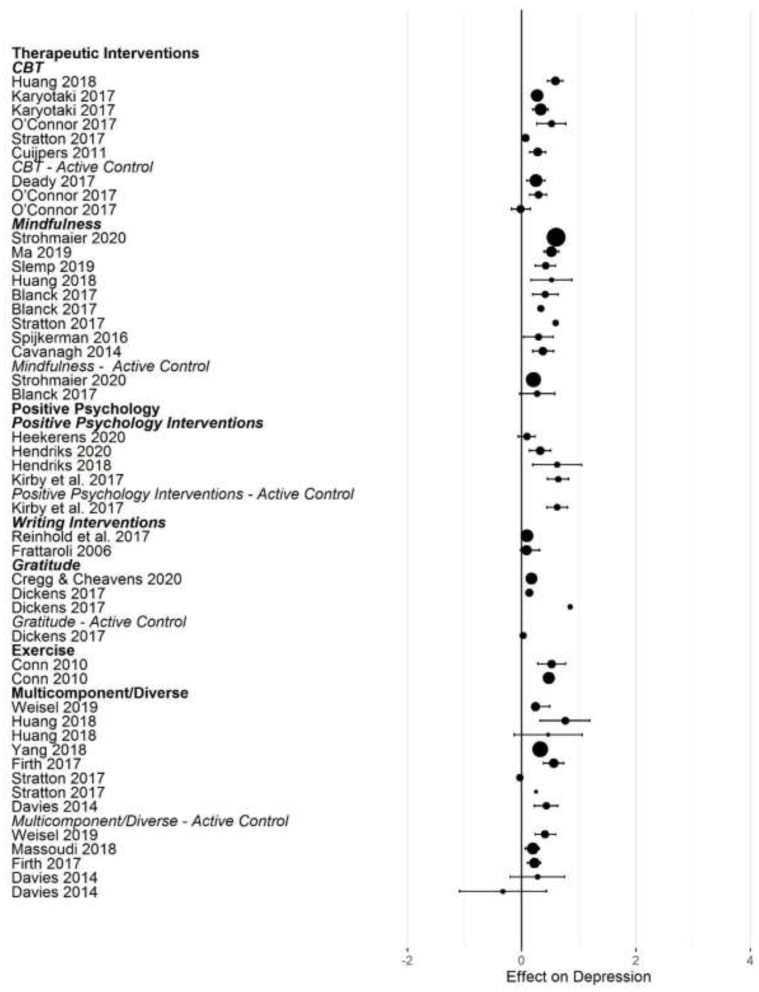
Forest plot of intervention effects on depression.

**Figure 4:**
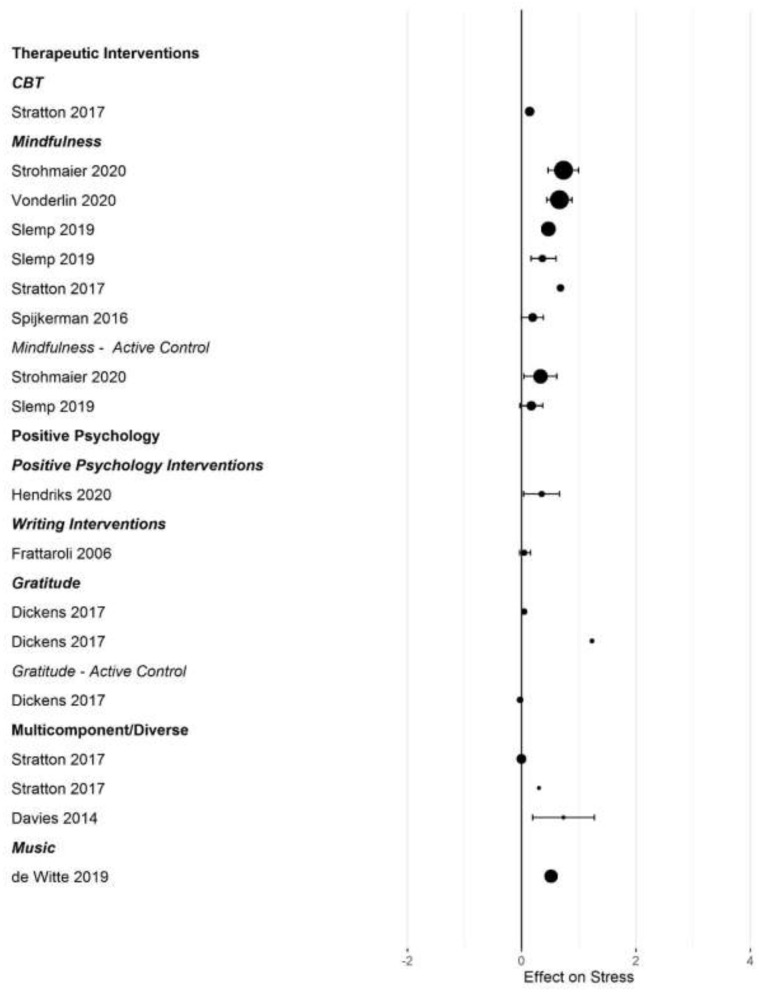
Forest plot of intervention effects on stress.

**Figure 5:**
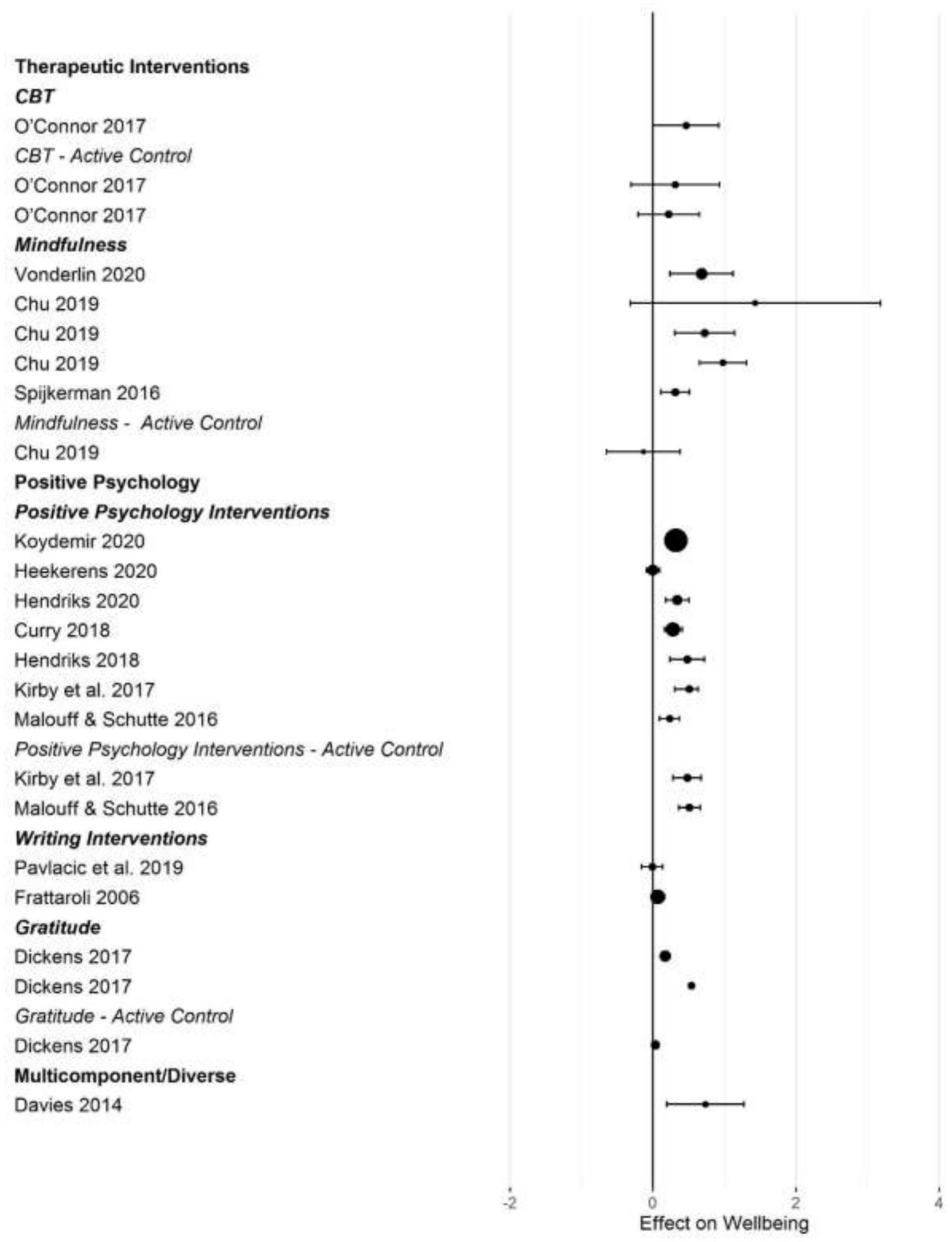
Forest plot of intervention effects on subjective well-being.

Figures 2–5 show the effect sizes and confidence intervals (if reported) from the meta-analyses (we converted *r* coefficients reported in Frattaroli, 2006 into *d*). We recoded effect sizes for anxiety, depression and stress so that positive numbers indicated a positive change (improvement) for the experimental group compared to the control group. As Figures 2–5 demonstrate, most meta-analyses showed an advantage of the intervention compared to the control group, but the type of control group appeared to impact the observed effect size.

After converting standardized mean differences to *z*-transformed *r*, the average effect size *r* was comparable across the four outcome variables, for anxiety *r* = 0.19 (95% CI: 0.14, 0.24; *k* = 31); depression *r* = 0.17 (95% CI: 0.13, 0.20; *k* = 45); subjective well-being *r* = 0.19 (95% CI: 0.13, 0.25; *k* =25), and stress *r* = 0.19 (95% CI: 0.11,0.26; *k* =17). Effect sizes computed for comparisons with active control groups yielded smaller effects, but this difference was only significant for depression (Q [1] = 5.70, *p* = 0.017, *R*^2^ = 0.096).

When examining differences between types of interventions, we used therapeutic approaches (CBT, ACT, and MBI) as the reference category. For positive psychology interventions, we separated gratitude and expressive writing interventions from other positive interventions. Due to the small number of effect size summaries available, we included activity-based interventions (exercise, music) with other/multicomponent interventions. For anxiety, we found a significantly larger effect for mixed, multicomponent and other interventions compared to CBT and ACT (*b* = 0.19 [95% CI: 0.05–0.55]; *p* = 0.009). For subjective well-being, we found a trend for mindfulness interventions to show larger effect sizes compared to CBT (*b* = 0.16 [95% CI: = 0.02 −0.34], *p* = 0.086). For depression, we first controlled for active control group comparisons (see the results reported above). Expressive writing exercises had a significantly smaller effect size on average compared to CBT/ACT based interventions (*r* = −0.15 [95% CI: −0.29 to −0.00], *p* = 0.017). We did not find statistically significant differences in the effectiveness of different interventions for stress.

## Discussion

Our rapid review of available meta-analyses demonstrated that there are a number of evidence-based self-guided interventions that can be used by individuals at home to manage depression, anxiety, stress, and well-being during stay-at-home orders, lockdown, and quarantine. Overall, self-guided interventions are better at improving psychological health compared to no intervention (e.g., waitlist controls) and, to some extent, active controls (e.g., comparable treatments). In particular, self-guided therapy-derived interventions (including CBT, ACT, and MBI), mindfulness-based practices, positive psychology interventions, and activity-based interventions (e.g., physical exercise, music listening) appear effective in reducing anxiety, depression, stress, and in increasing subjective well-being compared to both active and inactive control groups. However, dose effects were largely inconsistent. Therefore, we cannot recommend specific intervals or durations for any of the intervention categories. Baseline effects were largely absent, implying that even individuals with elevated stress or psychological problems can use these practices at home without supervision, however we strongly recommend contacting health professionals if an individual is experiencing distress.

The unique context created by social distancing and quarantine necessitates reflection on the way self-guided interventions might be used. Although expressive writing interventions showed effectiveness compared to control groups, effectiveness was consistently lower compared to CBT and ACT-based interventions. Expressive writing about concerns or worries (including detailed reflections of difficult or traumatic events) may not be appropriate without adequate clinical support or guidance (Reinhold et al., 2018), especially when acutely experiencing negative emotional symptoms. Hence, we do not recommend these exercises for individuals to perform unsupervised at this current time of elevated collective worry and distress (see Wang et al., 2020).

Overall, self-guided activities included in these meta-analyses appear effective, but not as effective as in-person or group-based interventions. Therefore, these activities can be useful as a first line of psychological support during stay-at-home and lockdown periods, but they could not and should not replace more guided clinical interventions (either via telehealth or once in-person sessions become available again). Given the current strain on the mental health system and the likelihood of further restrictions in the near future, it is important to provide widely available evidence-based practices to avoid negative collateral effects on mental health at the population level (see Brooks et al., 2020; Duan and Zhu, 2020). This review provides an overview of best-practice self-guided interventions conducted prior to the pandemic that can be recommended and implemented at large scale to help and support populations at risk of mental health problems. However, self-guided interventions need to be complemented by further investment and strengthening of traditional mental health care support.

At the same time, the review clearly highlights blind spots in our understanding of evidence-based practices. More highly controlled research on self-guided and home-based interventions is needed to inform public health decision-making during pandemics that require quarantine and social distancing over potentially long periods of time. What are the ideal levels of compliance for self-guided mental health interventions beyond an initial lockdown period and how can mental health be maintained? When should self-guided interventions first be implemented or recommended to populations at risk and how long should these practices be maintained after the immediate lifting of more restrictive lockdowns? What are the effects of repeated lockdowns: should recommended self-guided interventions be switched or rotated? The meta-analyses summarize studies that were not specifically geared toward evaluating interventions that are focused on home practices during lockdown.

We isolate three main limitations of the current evidence-base to guide future research. A first gap, especially in the current context of global pandemic, is the lack of attention to culturally transferable interventions. Most studies have been conducted with samples from high-income, highly educated, and mainly Western nations. Given the greater population density and living arrangements in non-Western environments, these conditions may make effective mental health interventions even more urgent. We need further national and international multi-center research that includes diverse groups of participants to better understand whether interventions developed for autonomous individuals socialized into societies that emphasize individuality and self-reliance are as applicable and as effective in more community oriented contexts (Smith et al., 2013). A second major concern of current distancing measures which we were unable to address here is the potential for a negative impact on social relationships. The current lockdown measures require greater interpersonal skills, both in terms of living together with others in closed spaces for extended periods of time as well as maintaining contact with others outside the immediate social “bubble.” The current evidence is clearly geared toward the individual as the focus of the intervention, with little emphasis on social relationships (although gratitude interventions might be the single major exception). Thus, we need more evidence of the effectiveness of social interaction interventions. A third limitation is that our evidence is based on interventions that were conducted prior to the current pandemic. It is unclear whether the effectiveness of self-guided interventions is equally effective under the specific conditions of a pandemic. The baseline effects that we report make us cautiously optimistic about the continuing effectiveness of these interventions, even in conditions of increased overall stress and anxiety. A meta-analysis of controlled studies during the current pandemic would be highly beneficial.

Finally, it is worth considering the broader role of psychologists in responding to the COVID-19 pandemic. A major concern for individuals, groups, organizations, and nations is the economic impact of the current pandemic. The mental health impact of quarantine is more dramatic for lower income groups (see Reynolds et al., 2008). The medium and long-term negative economic impact of COVID-19 on the larger population, and especially financially and economically more vulnerable populations also needs greater attention from psychologists. Psychologists need to collaborate with economists and others involved in economic decision-making to consider options to support people to upskill and create new employment opportunities which help to alleviate this financial worry.

In summary, the current evidence suggests that a number of self-guided interventions suitable for at-home practice during lockdown and physical distancing are effective to for improving mental health. Specifically, we recommend interventions based on cognitive behavioral therapy, mindfulness, and acceptance-based activities, selected positive psychology activities, physical exercise, and music as useful first-line mental health interventions. However, these activities are not as effective as in-person and group based therapeutic interventions, and so they should not replace clinician-guided interventions for individuals and groups in need. Many of these interventions are now available via smartphone and web-based applications. In order to provide broad access to such evidence-based interventions to mitigate the negative side-effects of social distancing measures, this article includes an online supplement with selected exercises and further information to help individuals cope with the mental health challenges of physical distancing and quarantine.

## Data Availability Statement

Publicly available datasets were analyzed in this study. This data can be found at: https://osf.io/fpx4s/.

## Author Contributions

RF conceptualized the study and wrote the first draft. RF, TB, JK, MZ, KR, AR, and LG conducted the literature search and data extraction. RF, JK, and TB conducted the analyses and created the visualizations. DW coordinated the activity material collation. DW, TN, BI, and MC collated and summarized the activity material. PM provided feedback and advice. All authors approved the final version.

## Conflict of Interest

The authors declare that the research was conducted in the absence of any commercial or financial relationships that could be construed as a potential conflict of interest.

## Appendix A

### Search Terms

(“Mental health intervention” OR “self therap^*^” OR “mindful^*^” OR “meditation” OR “yoga” OR “positive psychology” OR “gratitude” OR “journaling” OR “expressive writing” OR “low intensity exercise” OR “applied relaxation” OR “self-guided” OR “affective touch” OR “physical exercise” OR “social-media” OR “mindful eating” OR “creative tasks” OR “occupational therap^*^” OR “social media intervention” OR “mental health app” OR “well-being app” OR “smartphone intervention” OR “art therap^*^” OR “music therap^*^”) AND “meta-analy^*^”

A second search used these more specific search terms:

(“quarantine” OR “isolation” OR “social isolation” OR “confinement”)

AND “meta-analy^*^”

AND “pandemic.”
