# Supplementary material for: Rapid Review and Meta-Meta-Analysis of Self-Guided Interventions to Address Anxiety, Depression, and Stress During COVID-19 Social Distancing
Source: Front Psychol. 2020 Oct 28;11:563876. doi: 10.3389/fpsyg.2020.563876 (PMC7655981; doi:10.3389/fpsyg.2020.563876)
Supplement: Supplementary file 1 [file Data_Sheet_1.pdf]

Mental Health Strategies for Quarantine & Social Distancing

Material prepared by

Daniel Wegerhoff, Nguyễn Thị Bảo Trâm, Briar Irving, Megan Crystal, Johannes Karl, Ronald Fischer

This is a practical complement to a rapid review & meta-analysis submitted for publication, please cite the overall project as:

Fischer R, Bortolini T, Karl JA, Zilberberg M, Robinson K, Rabelo A, Gemal L, Wegerhoff D, Nguyen, TB, Irving B, Chrystal M and Mattos P (2020) Rapid Review and Meta-Meta-Analysis of Self-Guided Interventions to Address Anxiety, Depression, and Stress During COVID-19 Social Distancing. Front. Psychol. 11:563876. doi: 10.3389/fpsyg.2020.563876

Supporting data is available on OSF: https://osf.io/ac6b4/

Content

Introduction
Cognitive Behavioural Therapy Exercises
- Noticing Unhelpful Thinking Styles
- Perspective Taking Exercise
- Noticing Your Emotions
- Managing Strong Emotions
- Managing your Behaviours
- Activity Scheduling

Acceptance and Commitment Therapy Exercises
- Stage 1: Accepting Your Reactions and Being Present
  - Defusion
- Stage 2: Choosing Valued Action
  - Getting to Know Your Values
- Stage 3: Take Action
  - Goal Setting

Mindfulness Exercises
- Body Scan
- Mindful Eating

Positive Psychology Exercises
- Positive Writing Exercise
  - Three Good Things
  - Start Your Gratitude Journal

The Importance of Physical Exercise
Sleep Exercises
- Sleep Hygiene
- Sleep Scheduling

Sources

Introduction

Every person will experience worry, sadness, fear or stress at some point in their lives, they are natural human emotions. Sometimes however, these feelings can become overwhelming and difficult to deal with. This is especially common during uncertain and stressful times – such as a global pandemic – as we struggle to adapt and make sense of what is going on around us.

During such times it is important to have strategies that we can use to look after ourselves and regain a sense of control. In order to help, we have compiled a toolkit of strategies that you might like to consider.

We have searched thousands of studies and gathered insights and advice from world-leading experts to put together these activities. Some of them are based on ancient wisdom and philosophical practices from the oldest civilisations, others are derived from psychological and neuroscience research on how our brain and body work together.

Read through these activities and exercises. We have grouped them by major intervention approaches that psychologists may use. We will start off with some techniques and approaches that are used in modern psychology. Remember though, there is no one-size fits all, and not all of the exercises will necessarily feel like your cup of tea but feel free to give them a go! Some of them require working through lists and activity sheets; others will focus on your breathing and your body. Experiment with them and see how you feel with each set of exercises.

Disclaimer:
The information provided throughout this document is for general informational/educational purposes only. We do not provide any medical or health advice and recommend anyone experiencing medical or mental health difficulties to seek professional advice before using these resources. The contents of this document are not a substitute for professional medical advice, diagnosis, or treatment. Any actions taken based on the information provided within this document should be done so upon proper consultation with the appropriate professionals. The authors do not accept any liability for the use of or reliance on any information contained in this document. We do not guarantee that this information is correct, complete, reliable, or suitable for any purpose. For mental health and medical professionals, these resources should be considered an informative supplement to good practice, not a replacement. It is the responsibility of professionals to engage in competent, ethical, and informed practice. We do not recommend or endorse any specific professionals, tests, products, procedures, opinions, or other information that may be mentioned within the document or the sources provided.

Cognitive Behavioural Therapy

Cognitive Behavioural Therapy (CBT) is based on the theory that psychological distress can occur when we think and behave in unhelpful ways. Our thoughts and behaviours can influence each other as well as our emotions, feelings in our body, and our environment – so having unhelpful thoughts and behaviours can have a big effect on our lives. Here's a simple example of how these things may influence each other:

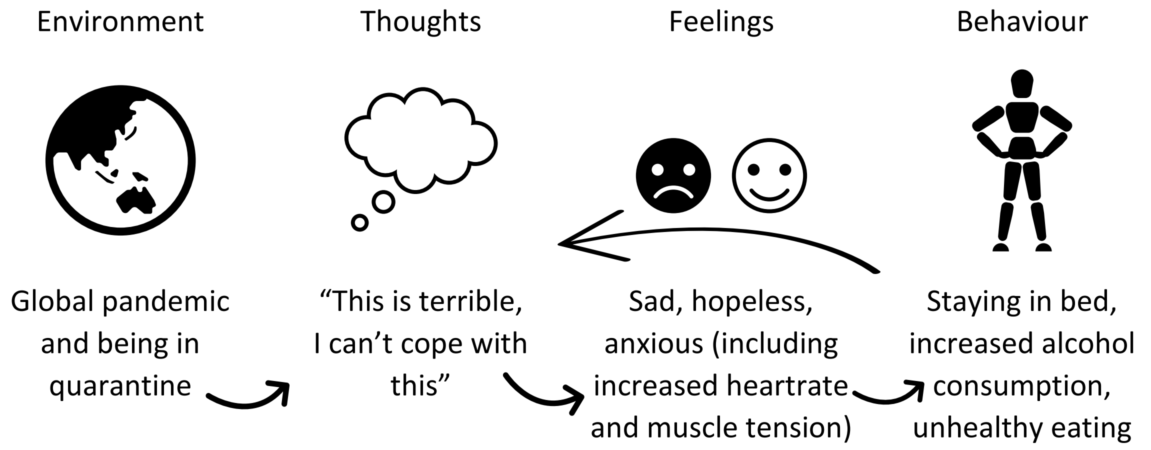

Therefore, if we can change unhelpful thoughts and behaviours, then we can change the distress they cause us. The exercises in this section are designed to help you understand your thought patterns, think differently, manage your emotions, and behave differently to help reduce distress.

Noticing Unhelpful Thinking Styles

The first step to changing how you think is to notice unhelpful thoughts. This can be hard because sometimes we're so used to thinking in a certain way that we don't realise it's making life difficult. Below is a list of common types of unhelpful thinking. Which ones do you experience most often?

- **Mental Filter:** Focusing on only one part of a situation and discounting other aspects. E.g., focusing only on the negative aspects of quarantine and not seeing positive aspects.
- **Jumping to Conclusions:** Assuming that we know what someone is thinking or what will happen in the future. E.g., "If I go outside, I'll catch Covid-19"
- **Personalisation:** Taking responsibility for things that go wrong even when we might be only partly responsible or not responsible at all. E.g., "If my family gets sick it'll be my fault for not keeping them safe".
- **Catastrophising:** Thinking of the worst possible outcome to a situation. E.g., "I will go crazy and have a mental breakdown if I have to stay in isolation for weeks".

- **All-or-Nothing Thinking:** Thinking in extremes and not considering less extreme alternatives. E.g., "The gym is closed so I can't do any exercise".
- **Overgeneralising:** Seeing a pattern based on one event. E.g., "I can never do anything right"; "no one ever helps me".
- **Minimisation:** Discounting the importance of something. E.g., "It doesn't matter that I feel bad because other people have it worse".
- **Emotional Reasoning:** When the way you feel impacts the way you view a situation, e.g., "I feel anxious, so something bad is going to happen".
- **Demands:** Thinking that we should/must do something or be a certain way. This isn't always unhelpful, e.g., "I should wash my hands after touching things in public spaces" is a helpful thought to have in the current situation. But sometimes demanding thoughts put us under undue pressure, e.g., "I should be coping better than I am".

Perspective Taking Exercise

We all see the world differently based on things like personality, culture, upbringing, and life experiences. Our perspectives influence the way we interpret different situations. This is why two people who experience the same event can react to it in very different ways.

Sometimes, our perspectives can cause us to have unhelpful thoughts, unpleasant emotions, and to behave in ways that end up making our lives worse. Therefore, practising taking other perspectives when a situation upsets us can help us change unhelpful thought patterns and improve our lives.

The following exercise can help you to identify your automatic interpretation of a situation and then prompts you to think about that situation in a variety of ways. Try break down the different parts of your automatic thought process by examining what might have happened during a recent situation:

- **Upsetting situation:** What happened, who was there, when and where did it happen?
- **Emotions and bodily sensations:** What physical sensations and emotions did you feel during and after that situation?
- **Automatic thoughts and interpretation:** What thoughts, images, and/or memories came to mind, how did you interpret these as they popped up or appeared in your mind? Think back to the "unhelpful thinking styles" exercise, did you do any of these?
- **Alternative perspective/compassionate response:** Can you think of alternative perspectives that describe what happened in that situation? What would this situation look like from another perspective (as if seeing the situation from an outsider's view)? How might a close friend or someone you admire interpret the situation? How might you look back at this situation in the future? Can you feel more compassionate towards yourself or others?

If you want, draw these out on a piece of paper.

Upsetting Situation

E.g., At home alone this evening, watched a news story about people getting sick with Covid-19.

Emotions and Bodily Sensations

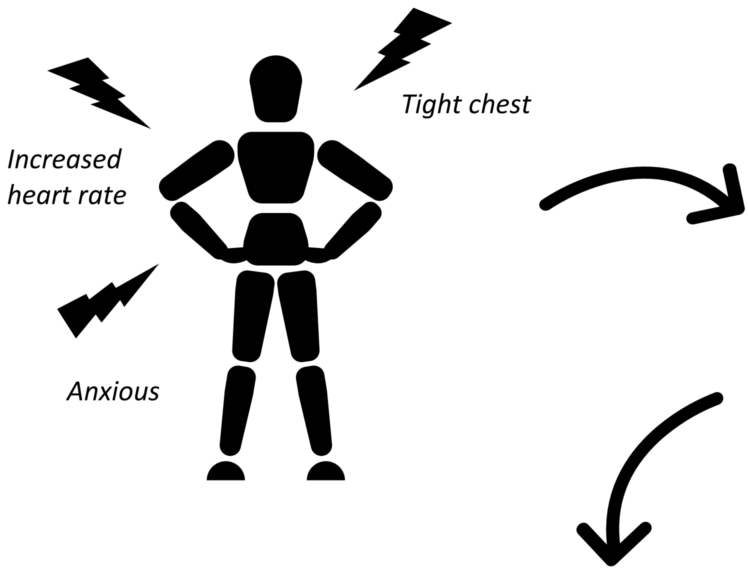

Automatic Thoughts and Interpretation

E.g., I'll get sick if I go outside; things are too scary, and I can't cope with it; what if someone I know dies from Covid-19? I worry so much, therefore I'm incapable of dealing with this.

Alternative Perspective/Compassionate Response

E.g., If I wash my hands often and stay away from other people, I'm less likely to get sick; I've been scared before and was able to cope; it's also possible that no one I know will die of Covid-19.

Noticing Your Emotions

Emotions are hugely important aspects of our lives because they motivate us to behave in ways that keep us safe, healthy, and fulfilled. Even unpleasant emotions such as anxiety, fear, and disgust are useful because they help keep us away from danger.

Emotions become problems for us when they're overwhelming and we don't know how to manage them effectively. The first step to managing emotions is to notice how we experience them and how they impact our lives. Consider the following questions to help you understand your emotions.

- What emotion do I feel the most often?
- What emotion do I feel the least often?
- Where in my body do I feel my emotions?
- What situations make me feel certain emotions?
- How do my emotions affect my behaviour – what do they make me do more or less of?
- Are the ways I act on my emotions helpful – do they take me closer to being the person I ideally want to be?
- How do I feel about my emotions?

Managing Strong Emotions

Behaviours that help us reduce unpleasant emotions in the short-term can make life worse in the long-term (e.g., drinking alcohol, doing drugs, over-eating, or avoiding unpleasant situations altogether). Therefore, it's important to learn ways to manage strong emotions that won't make things worse.

If you are feeling overwhelmed, you could try one or more of the following ideas to help you cope:

- Focus your attention on something else, like your breathing
- Try a body scan or relaxation exercise (we describe these in the mindfulness section)
- Watch a light-hearted movie, TV show, or online video
- Read a book
- Do household chores
- Talk or write about your feelings
- Do some exercises or stretches

Managing Your Behaviours

As we've mentioned, sometimes the things we do in response to our environment, thoughts, and emotions can end up making our situation worse. In this section, we will focus on what you can do if you're feeling low, sad, or depressed.

When we feel low, we become less active. The less active we are the fewer opportunities there are for positive and rewarding things to happen to us. The fewer rewarding things that happen

to us the lower our mood becomes. Behavioural activation – doing more rewarding behaviours – is one way out of this vicious cycle. It is a practical and evidence-based treatment for depression – especially when used in conjunction with other exercises.

Activity Scheduling

This is an exercise designed to help you fit more pleasant activities into your week and notice how they may affect your mood.

Step 1. Make a list of activities you find rewarding and can do given what isolation measures your government has enacted. Include as many activities as you can think of. Here are some examples to get you started:

- Cook your favourite meal
- Talk to a friend
- Do something nice for someone
- Listen to music
- Learn a new skill

Step 2. Choose some of your rewarding activities and put them in the Activity Scheduling Worksheet below. *Do not* try to fill in every square. Just try to add in more positive experiences than you're currently having, even if that's only one or two things. Trying to do too much too quickly can be unhelpful as it might be overwhelming, decrease your chances of success, and make you feel worse about yourself – start small and gradually increase your rewarding behaviours instead. Make sure to also put in activities that are your responsibility even if they're not necessarily enjoyable (e.g., chores, showering, brushing your teeth).

Step 3. Record how low you felt before and after the activity in the space provided. This will help you notice how behaviours affect your mood.

Activity Scheduling Worksheet

Add some rewarding activities into the white spaces provided in the schedule below. Be realistic about what you can achieve – *do not* fill in every box!

Before you do the activity, rate how low you feel on a scale from 0-10 (0 = extremely low, 10 = best you've ever felt) and write it in the space provided. Rate your mood again after you've done the activity. Write down what you noticed while doing the activity.

	Mon	Tues	Wed	Thur	Fri	Sat	Sun
8am-9am							
9am-10am							
10am-11am							
11am-12pm							
12pm-1pm							
1pm-2pm							
2pm-3pm							
3pm-4pm							
4pm-5pm							
5pm-6pm							
6pm-7pm							
7pm-8pm							
8pm-10pm							
10pm-12am							

Activity	Rating Before	Rating After	Comments
Have breakfast	*4/10*	*6/10*	*Felt more awake and less hungry afterwards.*

Acceptance and Commitment Therapy

The goal of Acceptance and Commitment Therapy (ACT) is to help individuals pursue meaningful and fulfilling lives by identifying their personal values and acting in a manner that aligns with these values. Painful experiences and unforeseen difficulties are an inevitability of life; therefore, ACT emphasises the importance of developing tools to dealing with such difficulties in a realistic and constructive manner. Specifically, this requires an individual to be able to distinguish between the factors within and beyond their control. By identifying these factors, a person is better positioned to prioritise those which are 'controllable', in order to act in a manner which aligns best with their key values.

In ACT, thoughts, memories, mental images, sensations, urges, and feelings are all considered examples of relatively uncontrollable factors; thus, individuals are encouraged to acknowledge and accept these experiences but to focus on their *responses* to such experiences (i.e., their appraisals of the thought/feeling, subsequent actions, etc.). This process is described in three key stages: (1) accepting your reactions; (2) choosing a valued action; and (3) taking action. Below is a short description of each stage along with some useful activities to help with each.

Stage 1: Accepting Your Reactions and Being Present

As we've discussed in the CBT section, sometimes the ways we react to events or even our own thoughts and emotions can make our situation worse. We can get into battles with our own thoughts and feelings – ruminating on them, trying to push them away, arguing with them, and generally spending time and energy on them instead of what makes our life rich and meaningful. Consider the following example:

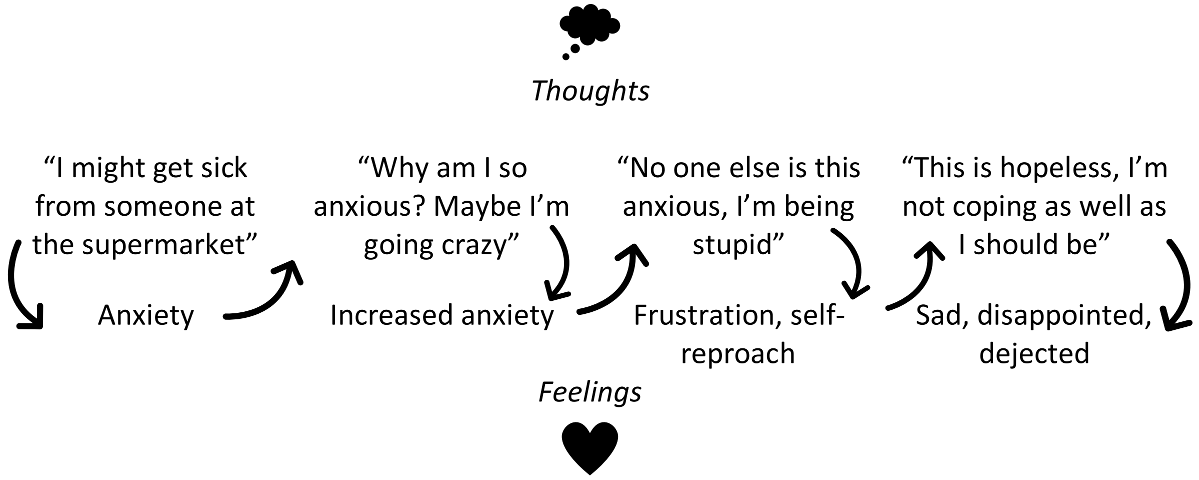

One goal of ACT is to help you stop getting stuck in unhelpful cycles like this one by teaching you ways of responding to thoughts and feelings other than struggling with, judging, and avoiding them. ACT encourages you to notice and accept your thoughts and feelings for what they are: normal human experiences that come and go in their own time.

Defusion
Imagine your difficult thoughts and feelings are written down on a piece of paper. When you're caught up in them, it's like holding that piece of paper right in front of your face – they obscure your view and make it hard for you to engage with the world around you. We call this "fusion" because you become fused with these experiences at the expense of meaningful aspects of your life.

No matter how hard we try, we can't stop unpleasant thoughts and feelings from occurring. In fact, the more we try to get rid of them, the more time and energy we spend on them and the more we get sucked into unhelpful chains of thoughts, feelings, and behaviours. The ACT response to fusion is "defusion". Defusion is like taking the paper from in front of your face and resting it on your lap; it's still there, but now it doesn't get in the way of you engaging with the world in a meaningful way.

Here are some defusion techniques you can try out next time you notice you're fused with a thought or emotion.

- **Write:** Write down your thought/emotion on a piece of paper. Try writing it many times in different fonts, colours, and sizes.
- **Label:** Simply label what you're experiencing as a thought, memory, emotion, sensation, urge, prediction, or plan. If it's a thought, you could see if it's any of those listed in the Unhelpful Thinking Patterns section of this resource.
- **Metaphors:** Try imagining your thoughts as leaves floating down a stream, clouds moving across the sky, or cars driving down the road. Notice how they eventually pass, even if they do so slowly or get stuck on the way.
- **Add Perspective:** Try adding the words "I'm having the thought that…" in front of your thought, e.g., "I'm having the thought that I'm useless". Next, add "I'm noticing that…" to the sentence so it then becomes "I'm noticing that I'm having the thought that I'm useless".
- **Mindfulness** is another defusion technique that we describe in more detail in the next section of this resource.

Stage 2: Choosing Valued Action

There is no 'one right way to live life'. In order to work towards living a meaningful and fulfilling life it is important to understand the values that make your life meaningful to you. Complete the following exercise to help clarify what values guide your life and what you want to stand for in the world.

Getting to Know Your Values
Look at each of these four areas of your life. Write down some values that you find important in each area. There may be some overlap between areas.

Some questions to help you think of values are:

- What type of person do you want to be?
- What do you want people to remember you for?
- What do you want your actions to be guided by?

Next, rate how satisfied you are with each domain in your life at the moment from 0-10 (0 = not at all satisfied, 10 = extremely satisfied).

**Life Domains**	**My Values**	**Satisfaction Rating**
Work/Education/Personal Development	*E.g., Creativity, perseverance*	
Social Relationships	*E.g., Compassion, fairness*	
Health and Spirituality	*E.g., Connectedness, physical fitness*	
Leisure and Recreation	*E.g., Humorous, adventurous*	

Stage 3: Take Action

Even in tough situations, you can use your values to give meaning and purpose to your life and guide your behaviours. For example, even in social isolation, someone who values kindness can find ways to be kind; they could send a message to a friend, speak kindly to themselves, or practice social distancing to help stop others from getting sick. Taking valued action isn't always easy, but it helps us become the people we truly want to be.

Goal Setting

Values are guiding principles in our lives, like the directions on a compass. Goals on the other hand are things we can accomplish or check off a to-do list. This exercise helps translate your values into meaningful action.

Step 1. Choose a value you want to focus on for this exercise. You might want to focus on a value you haven't been acting on as much as you'd like.

Step 2. Write down one goal that you would like to achieve that would help you act in line with your values.

Step 3. Make sure your goal is SMART. SMART goals are:

Specific: outline exactly what you want to achieve
Measurable: you will be able to know when it's been achieved
Achievable: relates to something you can directly and clearly influence
Realistic: you have the time, resources, and ability to achieve it. It isn't so ambitious that you're unlikely to achieve it
Time-framed: Has a specific date or time period that it can realistically be achieved in so it can't be put off indefinitely.

Step 4. Identify potential barriers that might get in the way of you achieving your goals and brainstorm solutions to these.

**Value**	*E.g., Novelty*
**SMART goal**	*E.g., Cook a new meal once a week during isolation restrictions.*
**Barrier(s)**	*E.g., Some ingredients may be out of stock at the supermarket; must keep shopping trips to a minimum to reduce likelihood of Covid-19 transmission*
**Solution(s)**	*E.g., Choose recipes that can be easily modified if an ingredient isn't available; plan meal before weekly grocery shop so no need to go twice*

Mindfulness Exercises

Mindfulness is about paying attention to what is happening in the present moment and attending to the details of each moment without judgement. The practice of mindfulness is based on ancient spiritual practices in South and Southeast Asia and has been used by people for millennia to regulate their emotions. Modern research has demonstrated that practicing mindfulness can indeed promote wellbeing and help us to feel less stressed, anxious, and depressed. As you might have seen, the various activities on the previous pages often require you to pay attention to what you are doing and be present in your current thoughts and feelings. In the following exercises, we will provide a few more of these activities that can help you to practice calming yourself and focusing on the here and now.

Body Scan

This exercise helps you reconnect with your body. Try to keep your focus on your body. It's likely that your mind will come up with other thoughts, plans, worries, or memories. That's okay, just notice when your mind wanders and gently bring it back to focus on the task.

Begin in a lying down position on your back, with your legs extended, feet hip-width apart. If lying down isn't possible, feel free to do this sitting down in a comfortable chair. Position your arms by your sides with palms facing up. Focus your awareness slowly, deliberately, and systematically throughout the body. This can be done either from toe to head or head to toe. Focus on sensations, emotions, and thoughts about each part of the body as they arise.

Mindful Eating

In this exercise, you'll be guided through eating something mindfully. Choose a small snack that you'll be able to hold, such as a piece of fruit, chocolate, candy, or a cracker. Make sure you wash your hands before this exercise if you've recently been in a public space.

- **Holding:** First, take the snack and hold it in the palm of your hand or between your finger and thumb.
- **Seeing:** Take time to really focus on your food. Focus your full attention on what this food looks like. Notice its colour; is it one colour or many colours? Notice the way the light hits it; does one side have more light on it than another? Notice its shape and where it has curves, edges, and flat surfaces.
- **Touching:** Use your fingers to explore the texture of your food. You can do this with your eyes closed if that helps you focus on texture.
- **Smelling:** Hold the snack under your nose. Breathe in any fragrance that comes from your snack. Notice if anything happens in your mouth or stomach as you do this.
- **Placing:** Now slowly bring the snack up to your lips. Gently place the snack in your mouth if it's small enough or slowly take a bite so there's a piece of food in your mouth. Notice any urges to chew, but don't start chewing now. Spend a few moments focusing on what it feels like having this food in your mouth.
- **Tasting:** Prepare to chew your food, noticing where in your mouth it needs to be for chewing and how it gets there. Then slowly take one or two bites and notice what happens

to the taste and texture of the food. Notice any sweetness, saltiness, bitterness, or spiciness.

- **Swallowing:** When you're ready, try to notice the urge to swallow before actually swallowing the food.
- **Following:** See if you can feel what you swallowed moving down into your stomach, and broaden your attention to you whole body and how it feels having done this

Positive Psychology

Positive psychology is a movement within psychology that emphasizes the positive aspects in people's lives and aims to help individuals grow by emphasising optimism, resilience and positive strength.

Positive Writing Exercises

Writing exercises can be a useful way of taking time to reflect on a number of aspects of your life. Whether its spending a few extra moments re-enjoying positive experiences, contemplating the kind of person you would like to be, practising forgiveness, or expressing gratitude for the good things in your life – writing can be a great way of reflecting on difficult experiences and taking time to appreciate moments that may otherwise go unnoticed. Below are two writing exercises that you might like to try. The first one, you can do every day. Spend five minutes each evening and remember what happened each day and focus on the positive. The second, you can do a few times a week. Don't worry if you forget them, just get back to them when you can. You don't need to spend much time on them, take 15 min in the evening to reflect on the events of the day or since you last wrote a note. Jot down your thoughts on a piece of paper or on a document on your phone or computer. If you spend a lot of time on your phone or computer, maybe take the opportunity and write your thoughts on a piece of paper.

Three good things

This is a very simple but effective exercise for helping you reflect on the positive aspects of your life. Each evening, write down three events from the day that seemed positive, and why they felt that way. Write as little or as much as you want. Concentrate on the positive things. This is an exercise you can do every day.

Start your gratitude journal

Appreciate the good things about someone important in your life. Focus on a partner, close friend or family member and take time to think through the following questions listed below. Keep a record so that you can review it.
A few important suggestions before you start:

- **Be specific:** The more specific you are the better.
- **Focus on people:** Focusing on people for whom you are grateful is more effective than writing about things.
- **Deeper is better:** Elaborating in detail about a particular thing for which you are grateful is more beneficial than making a shallow list of many things.
- **Savour surprises:** Recording events that were unexpected or surprising tends to elicit stronger feelings of gratitude.
- **Set your intention:** Your motivation to be happier plays an important part in your development of gratitude. Like many things in life, the more you engage the more you will get out of it – if you just go through the motions it will be less effective.

- **Be consistent:** Once you have set your intention to record things you are grateful for, honour your intention by sticking to it. Set aside 15 minutes at a specific time of day to complete your journal.
- **Don't overdo it:** For this kind of writing, writing occasionally (1-3 times per week) has been shown to be more effective than writing daily.

For each person or event, try to note down specific examples. If it helps you, try to answer these questions:

- What drew you to this person (e.g., your partner, friend) when you first met?
- What things have you really enjoyed doing together during your relationship?
- What things do you really appreciate about them right now?
- What are their strengths?

Then (and this is the important bit!), when you're with that person or you talk to that person online take the time to notice and acknowledge these things – their strengths, the things they do that you really appreciate, the happy times you've shared together and so on.

> "I really love it when you… "
> "You're so good at… "
> "Seeing you do… reminds me of that fantastic day when we… "

Physical Exercise

Regular physical exercise plays an important role in improving and maintaining not only your physical fitness but also your overall well-being including mental health, energy levels, sleep, and mood.

It is generally recommended that we get 2.5 hours of moderate-intensity aerobic exercise or 1.25 hours of high-intensity aerobic exercise per week.

There are many excellent resources available online that go through simple exercises.

There are also excellent resources for yoga, which can be more meditative or more physically activating, depending your choices. Use these resources!

Important: Make a commitment to set aside some dedicated time on specific days. Develop a routine for your exercise.

Sleep Exercises

Sleep is the body's natural way of resting in order to physically and mentally prepare for the next day. Getting good quality sleep at the right times is a necessary and central component of good health and well-being. Unfortunately, major disruptions – such as those that have come with the Covid-19 pandemic – can severely impact our ability to get good quality and enough sleep.

The current environment creates many possible sources of sleep problems, there are disruptions to our usual routines; we spend more time in front of screens; we may experience increased anxieties, worries, and stresses due to the virus and all the news around it; the effects of isolation; not being able to exercise as usual; and changes to our diet. All of these factors can disrupt our sleep routines, making us more tired during the day and keeping us up at night.

Not getting a good night's sleep can also 'feed back' into these problems, creating a negative spiral that can be difficult to change (e.g., feeling tired or exhausted can negatively impact our mood and make regular challenges feel more difficult than usual, which might make us worry more and therefore make it harder to sleep the next evening).

It is also important to be aware that there are a lot of strategies we might want to use to try to fix our sleeping that might actually be doing more harm than good. For example, we might be spending more time in bed to catch-up on lost sleep (i.e., going to bed earlier, taking naps, sleeping in); trying to force sleep; or watching videos and Netflix to help us to fall asleep. However, these activities are likely to *increase* sleep problems instead of decreasing them. To help you a get a good night's sleep, here are some evidence-based sleep-strategies and some key exercises to use to get control back over your sleep.

Improving Sleep Hygiene

Many people engage in practices that may be counter-productive to getting a good night's sleep. Sleep hygiene or "good sleep habits" are things you can do to help you to have a good night's sleep. These habits should be done in conjunction with other sleep activities such as sleep scheduling (which we describe below). The key idea is to adjust your lifestyle habits and environment to facilitate better sleep.

Winding down

Here are some preparatory actions that you can do to help you fall asleep more easily:

- Try to establish a relaxing routine in the lead up to bedtime (e.g., reading a book, practicing mindful relaxation techniques).
- Avoid stimulating activities (e.g., loud music, video games, electronic devices) at least 30 minutes before bedtime
- Limit bright lights in the evenings.

- Reduce your fluid intake before bedtime. It is important to stay hydrated, but it is better to drink during the day rather than in the evening. Especially, limit caffeine (coffee, energy drinks, cola) from the afternoon onwards and alcohol before bed.
- Avoid eating before bedtime. If you get hungry try a light snack but be careful to avoid anything with too many sugars.

Associating your bed with sleep

- It is important that your body recognises your bed as a place for sleep. Don't go to bed unless you are tired and ready to sleep.
- If you go to bed but are unable to fall asleep after 20 minutes, get out of bed and do a relaxing activity until you are ready to try again.
- Reduce noise in your bedroom and create a relaxing atmosphere.
- Keep your bedroom at a comfortable, cool temperature.

Eat healthy & do regular exercise

Healthy diet and regular exercise during the day can help your body feel ready for sleep in the evenings. However, as noted above, don't eat large meals in the evening and make sure not to exercise too close to your bedtime.

Sleep Scheduling

Many people with sleep problems tend to try and spend more time in bed to catch up on lost sleep. However, spending more time in bed does not mean you spend more time asleep and can negatively impact your ability to fall and stay asleep.

A good strategy for better sleep is to *limit* the amount of time you spend in bed to 'sleep time' only. Reducing time spent in bed to your 'average time spent asleep' helps to reset your body's expectations about sleep and work towards making sleep more efficient (i.e., improving sleep *quality*).

Step 1. Start a sleep diary. A sleep diary will help you track how much you are sleeping, as well as how much time you are spending in bed awake. Each morning – for one week – record the following about your previous night's sleep:

- What time did you go to bed?
- What time did you fall asleep?
- Did you wake up in the night, how long for?
- What time did you (last) wake up in the morning?
- What time did you get out of bed?

Step 2. Once you have kept a sleep diary for a week, calculate the average time you have spent *asleep* across these days. This can be done by calculating:
(total number of hours asleep) / (number of nights) = average time spent asleep.

Once you have these hours, put your average hours slept into a sleep restriction calculator. A good example is: https://insomniacoach.com/sleep-restriction-therapy/
The calculator will calculate the time you are now allowed to *spend in bed in total*.

Step 3. Use the time you calculated in the previous step to calculate your 'sleep window'.

To calculate your sleep window:
1. Work out when you want/need to be out of bed the next morning
2. Subtract the amount of time you are allowed to *spend in bed in total* from this time
3. This will give you the start of your sleep window, i.e., the point at which you try to get to sleep.

Step 4. Put your new sleep window into practice for the next week. Make sure you only go to bed when you are sleepy and ensure you are out of bed once your sleep window has ended. Keep recording your sleep diary during this week

Step 5. After a week of putting your new schedule into practice, it is now time to review and update your sleep window to make sure you are getting the most out of it. This is done through calculating your *sleep efficiency*.

Sleep efficiency is the amount of time you spend asleep out of the total time you spent in bed. More efficient sleep means you spend less time in bed awake and more time actually sleeping.

It can be difficult to figure out your sleep efficiency. Use the sleep calculator from the link above (or other tools) to help you.

What was your sleep efficiency?

(1) Less than 85%

It is generally recommended that if your sleep efficiency is less than 85% then you should reduce your sleep window by 15 minutes for the next week (i.e., go to bed 15 minutes later). However, make sure you never reduce your sleep window to less than 5.5 hours, this should be considered the bare minimum sleep window.

Once you have done this for one week and recorded that week's sleep diary, recalculate and review your sleep efficiency again.

(2) 85% or more

You seem to be sleeping relatively efficiently, for the next week try increasing your sleep window by 15 minutes (i.e., go to bed 15 minutes earlier). For some who are sleeping at 95% efficiency or greater, you might want to try increasing your sleep window by 30 minutes, but only if your sleep efficiency is consistently at this point.

Once you have recorded that week’s sleep diary, recalculate and review your sleep efficiency again.

Take home notes: Sleep scheduling will help you to train your body to be ready to fall asleep and wake up at specific times. Hopefully over time, you should gradually start to see improvements in the quality and efficiency of your sleep. It can take a little while to see improvements but stick at it and try to be strict on yourself when you put the times into practice!

Sources

We compiled, adapted and copied these activities from a number of sources. Here are some key sources. Feel free to consult these original resources because there is often additional material that might be interesting for you.

CBT

Centre for Clinical Interventions. (2019). Looking after yourself: Depression. Retrieved from https://www.cci.health.wa.gov.au/Resources/Looking-After-Yourself/Depression

Psychology Tools. (n.d.). A guide to emotions. Retrieved from https://www.psychologytools.com/self-help/a-guide-to-emotions/

Psychology Tools. (n.d.). CBT daily activity diary. Retrieved from https://www.psychologytools.com/resource/cbt-daily-activity-diary/

Selva, J. (2020). Behavioural activation: Behavioural therapy for depression treatment. Retrieved from https://positivepsychology.com/behavioural-activation-therapy-treating-depression/

Therapist Aid. (n.d.). Behavioral activation guide. https://www.therapistaid.com/therapy-guide/behavioral-activation-guide

Vivyan, C. (n.d.). Cognitive behaviour therapy. Retrieved from https://www.getselfhelp.co.uk/cbt.htm

Whalley, M. (2019). Unhelpful thinking styles: Cognitive distortions in CBT. Retrieved from https://www.psychologytools.com/articles/unhelpful-thinking-styles-cognitive-distortions-in-cbt/

Whalley, M., & Kaur, H. (2020). Living with worry and anxiety amidst global uncertainty (UK English). Retrieved from https://www.psychologytools.com/

ACT

Ackerman, C. E. (2020). How does acceptance and commitment therapy (ACT) work? Retrieved from https://positivepsychology.com/act-acceptance-and-commitment-therapy/

Harris, H. (n.d.). ACT mindfully: Free resources. Retrieved from https://www.actmindfully.com.au/free-stuff/

Harris, R. (2006). Embracing your demons: An overview of acceptance and commitment therapy. *Psychotherapy In Australia*, *12*(4) 70-76. Retrieved from https://search.informit.com.au/

Harris, R. (2020). FACE COVID. Retrieved from https://www.actmindfully.com.au/

Hayes, S. C., Luoma, J. B., Bond, F. W., Masuda, A., & Lillis, J. (2006). Acceptance and commitment therapy: Model, processes and outcomes. *Behaviour Research and Therapy, 44*(1), 1-25. https://doi.org/10.1016/j.brat.2005.06.006

Vivyan, C. (n.d.). ACT: Acceptance and commitment therapy. Retrieved from https://www.getselfhelp.co.uk/act.htm

Mindfulness

Ackerman, C. E. (2020). 22 mindfulness exercises, techniques & activities for adults (+ PDF's). Retrieved from https://positivepsychology.com/mindfulness-exercises-techniques-activities/

Greater Good in Action. (n.d.). Raisin meditation. Retrieved from https://ggia.berkeley.edu/practice/raisin_meditation

Kabat-Zinn, J. (2005). *Coming to our senses: Healing ourselves and the world through mindfulness*. New York: Hyperion.

Shapiro, S. L., & Carlson, L. E. (2009). *The art and science of mindfulness: Integrating mindfulness into psychology and the helping professions*. American Psychological Association. https://doi.org/10.1037/11885-000

Positive Psychology

Action For Happiness. (n.d.). Happiness action pack. Retrieved from https://www.actionforhappiness.org/media/80216/happiness_action_pack.pdf

Chowdhury, M. R. (2020). 19 best positive psychology interventions + how to apply them. Retrieved from https://positivepsychology.com/positive-psychology-interventions/

Diebel, T., Woodcock, C., Cooper, C., & Brignell, C. (2016). Establishing the effectiveness of a gratitude diary intervention on children's sense of school belonging. *Educational and Child Psychology, 33*(2), 117–129.

Emmons, R. A., & McCullough, M. E. (2003). Counting blessings versus burdens: An experimental investigation of gratitude and subjective well-being in daily life. *Journal of Personality and Social Psychology, 84*(2), 377–389. https://doi.org/10.1037/0022-3514.84.2.377

Esposito, L. (2014). Ten positive psychology practices for boosting happiness. Retrieved from https://www.psychologytoday.com/us/blog/anxiety-zen/201409/ten-positive-psychology-practices-boosting-happiness

Killen, A., & Macaskill, A. (2015). Using a gratitude intervention to enhance well-being in older adults. *Journal of Happiness Studies: An Interdisciplinary Forum on Subjective Well-Being, 16*(4), 947–964. https://doi.org/10.1007/s10902-014-9542-3

Neff, K. (n.d.). Self-compassion. Retrieved from https://self-compassion.org/exercise-6-self-compassion-journal/

Seligman, M. E. P., Rashid, T., & Parks, A. C. (2006). Positive psychotherapy. *American Psychologist, 61*(8), 774–788. https://doi.org/10.1037/0003-066X.61.8.774

Physical exercise

World Health Organization. (2010). *Global recommendations on physical activity for health*. Retrieved from https://www.who.int/dietphysicalactivity/publications/

Sleep

Reed, M. (2019). Sleep restriction therapy for insomnia. Retrieved from https://insomniacoach.com/sleep-restriction-therapy/?

Sleep Education. (n.d). Healthy sleep habits. Retrieved from http://sleepeducation.org/treatment-therapy/healthy-sleep-habits/introduction

Sleepwell. (2020). CBTi components. Retrieved from https://mysleepwell.ca/cbti/cbti-components/

Stanford Health Care. (n.d.). Cognitive behavioral therapy for insomnia (CBTI): Sleep restriction. Retrieved from https://stanfordhealthcare.org/medical-treatments/c/cognitive-behavioral-therapy-insomnia/procedures/sleep-restriction.html
